# PD-1 inhibitors in advanced esophageal squamous cell carcinoma: a survival analysis of reconstructed patient-level data

**DOI:** 10.3389/fphar.2024.1408458

**Published:** 2024-07-18

**Authors:** Chunyan Yan, Wenxiu Cao, Jianghua Li, Lei Zhang, Ruigang Diao

**Affiliations:** ^1^ Department of Pharmacy, Yantai Yuhuangding Hospital, Yantai, China; ^2^ Yantai Municipal Government Hospital, Yantai, China

**Keywords:** PD-1 inhibitors, esophageal cancer, immunotherapy, first-line therapy, survival analysis

## Abstract

**Background:**

Recently, a sum of trials of programmed cell death-1 (PD-1) inhibitors combined with chemotherapy have shown excellent efficacy compared to chemotherapy alone in patients with previously untreated, advanced esophageal squamous cell carcinoma (ESCC). However, there is no head-to-head comparison and consensus on which immunotherapy regimen results in better survival outcomes. This study aimed to evaluate the survival efficacy of various PD-1 inhibitor–based therapies in the first-line treatments for patients with advanced ESCC.

**Methods:**

Data collected prior to 31 July 2023 were searched in the PubMed, Cochrane Library, Embase, Medline, and Web of Science databases. Overall survival (OS) and progression-free survival curves were pooled using the MetaSurv package. Survival data were compared by reconstructed individual patient data.

**Results:**

A total of 4,162 patients and seven randomized controlled trials were included. After synthesizing, PD-1 inhibitors prolonged median OS from 11.3 months (95% CI (confidence interval) 10.7–11.7) to 15.6 months (95% CI 14.7–16.3). Based on reconstructed patient-level data, the toripalimab, tislelizumab, and sintilimab group achieved the longest OS, whereas the sintilimab and tislelizumab group had the lowest risk of recurrence than other treatments. In patients with a combined positive score of ≥10, sintilimab had better OS efficacy than pembrolizumab (HR: 0.71, 95% CI: 0.52–0.96). In terms of tumor proportion score of ≥1%, camrelizumab, nivolumab, and toripalimab showed proximate survival benefits in both OS and progression-free survival.

**Conclusion:**

PD-1 inhibitor combined with chemotherapy significantly improved the survival time of patients with advanced ESCC. Toripalimab, tislelizumab, and sintilimab plus chemotherapy showed the best OS benefit. Longer progression-free benefits might be generated from adding tislelizumab and sintilimab to chemotherapy. Sintilimab was strongly recommended for patients with high programmed cell death–ligand 1 abundance.

**Systematic Review Registration:**

[https://www.crd.york.ac.uk/PROSPERO/], identifier [CRD42024501086].

## 1 Introduction

Esophageal cancer is one of the most common malignant tumors worldwide, with 604,000 new cases and 544,000 deaths, according to the Global Cancer Statistics 2020 (Sung et al., 2021). Esophageal squamous cell carcinoma (ESCC) is the predominant histological subtype of esophageal cancer (Arnold et al., 2020). China is a high-incidence area for ESCC, and although the incidence and mortality rates are on a downward trend, it remains a major malignant tumor threatening the health of our residents (Li et al., 2021). Presently, platinum-based chemotherapy is a commonly used regimen for advanced esophageal squamous carcinoma, but the prognosis remains poor (An et al., 2023). Therefore, there is an unmet demand for new therapeutic agents for advanced or metastatic ESCC.

Immune checkpoint inhibitors (ICIs) have significantly changed the treatment landscape for ESCC. Between 2019 and 2020, KEYNOTE-181, ATTRACTION-3, and ESCORT studies focusing on Chinese patients with ESCC have successively met the primary point in the second-line treatment (Kato et al., 2019; Huang et al., 2020; Kojima et al., 2020), which significantly improved overall survival (OS) of patients with advanced ESCC. Moreover, in 2021, KEYNOTE-590, the first global multicenter, Phase III clinical trial in the first-line treatment of advanced ESCC released that pembrolizumab plus chemotherapy provided superior overall survival benefits *versus* placebo plus chemotherapy (median OS: 12.6 months vs. 9.8 months; HR, 0.72; 95% confidence interval (CI), 0.60–0.88; Sun et al., 2021). In the following years, CheckMate-648 (Doki et al., 2022), JUPITER-06 (Wang et al., 2022), ORIENT-15 (Lu et al., 2022), and ASTRUM-007 (Song et al., 2023) published results successively, which confirmed that PD-1 inhibitors plus chemotherapy produced promising antitumor activity when compared with mono-chemotherapy.

Based on the survival benefit, the National Comprehensive Cancer Network recommended PD-1 inhibitor–based therapy as one of the first-line regimens. However, among numerous PD-1 inhibitors, physicians and patients may not be able to determine which may be more suitable for patients with ESCC in clinical treatment (Sun et al., 2021; Doki et al., 2022; Lu et al., 2022; Wang et al., 2022; Song et al., 2023) without direct head-to-head trial.

In our study, we employed a new, indirectly comparable method of survival analysis by combining or reconstructing KM curves (Liu et al., 2021; Messori, 2021). Moreover, we performed survival analyses in subgroups of different programmed cell death–ligand 1 (PD-L1) statuses, based on the CPS (combined positive score) and TPS (tumor proportion score). With this comparison of survival analyses, we aim to provide valuable survival information and give rational recommendations for the choice of treatment options in this field.

## 2 Methodology

### 2.1 Literature search

The protocol for this review was registered with PROSPERO (CRD42024501086). We conducted a systematic literature search through PubMed, Web of Science, Embase, Cochrane CENTRAL, and Medline on 31 July 2023, to identify Phase III clinical randomized controlled trials eligible for survival analysis. Search terms included “advanced esophageal cancer” or “advanced ESCC,” “immunotherapy” or “PD-1,” “chemotherapy,” “platinum,” “clinical trials” or “randomized controlled trials,” and “first-line.” Additionally, to identify more eligible trials, further searches of the bibliographies of the studies obtained after screening were manually performed. All search timeframes were from library construction to 1 June 2023.

### 2.2 Inclusion and exclusion criteria

The study was conducted based on the Preferred Reporting Items for Systematic Reviews and Meta-Analyses statement (Shamseer et al., 2015) by two independent authors. Inclusion criteria included the following: 1) patients with untreated advanced ESCC; 2) patients in the treatment group receiving PD-1 inhibitors plus platinum-containing two-agent chemotherapy; 3) Phase III placebo-controlled randomized clinical trials; and 4) Kaplan-Meier curves for OS and PFS reported for the primary or updated time-to-event data.

### 2.3 Data extraction and heterogeneity assessment

Specific treatment regimens, dosing cycles and doses, Kaplan–Meier curve, and the number of at-risk patients for each interval of time were derived from the included clinical trials. The survival probabilities of the curves were extracted with GetData Graph Digitizer. The statistical significance of heterogeneity was assessed by *I* ^2^ and *H* values (Higgins and Thompson, 2002). Predefined subgroup analyses were performed using the Meta package of R software based on sex, age, race, ECOG PS (Eastern Cooperative Oncology Group performance score), disease status at trial entry, and chemotherapy.

### 2.4 Statistical analysis

The OS and PFS curves from the seven studies were combined using the MetaSurv package in the R software (version 4.2.3; Combescure et al., 2014), thus directly reflecting the overall efficacy of anti-PD-1 inhibitors combined with chemotherapy in the first-line treatment of patients with advanced ESCC. Individual patient time-to-event data of each Kaplan–Meier curve was digitalized using the R package IPDfromKM (Messori, 2021), and the KM curves were redrawn based on the reconstructed individual data for efficacy comparisons between PD-1 inhibitors. HR and 95% CI were calculated using Cox statistics. Pooled raw HR value from original trials was calculated by using the Meta package of R software. The risk assessment was analyzed by using the Cochrane risk of bias tool. Sensitivity analysis was conducted by using the one-by-one elimination method to test the robustness of merging outcomes.

## 3 Results

**FIGURE 1:**
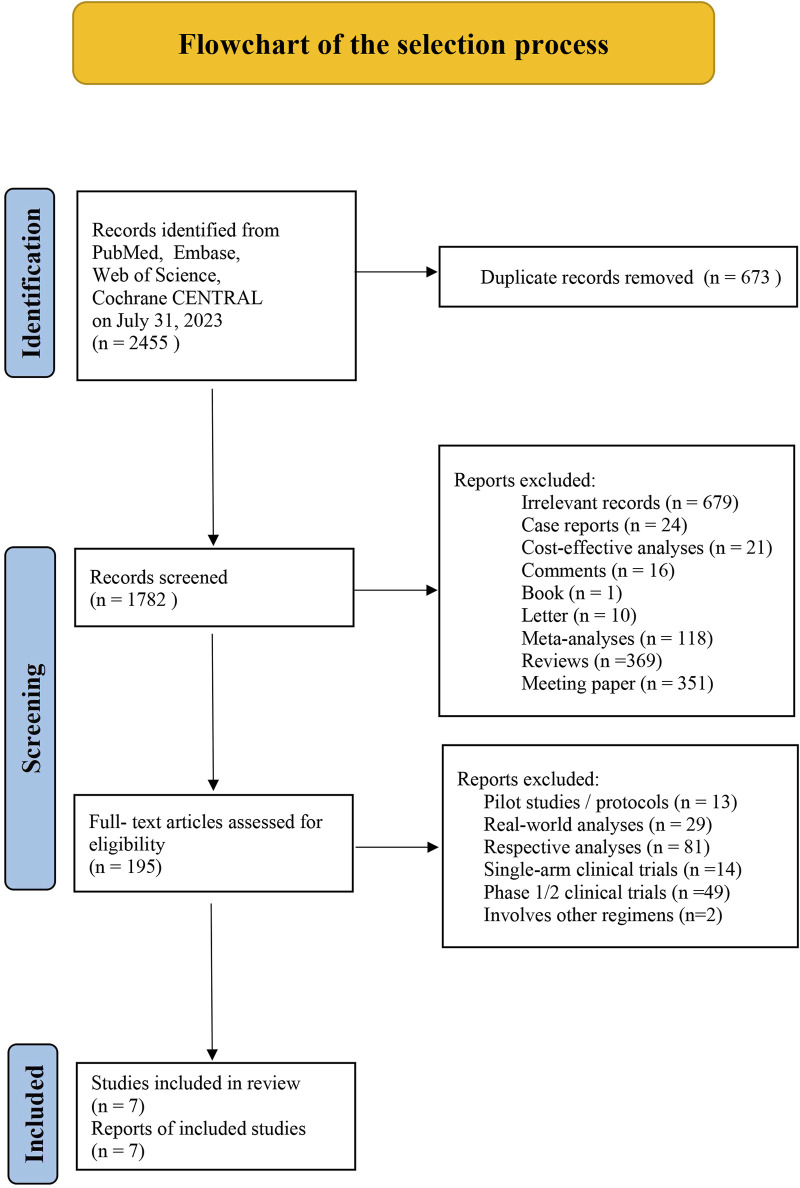
Flowchart of the selection process.

The literature selection flowchart for this study is presented in Figure 1. A total of 2,455 records were identified. Before the screening, 673 duplicate records were removed. After reviewing by title and abstract, 1,587 records were eliminated as they did not match the initial requirements. Afterward, 195 articles were assessed in full-text evaluation, and seven records were eligible for the next step of survival analysis in this study. The characteristics of eligible studies included are shown in Table 1.

**TABLE 1:** Baseline characteristics of the enrolled trials.

Study (year)	Author	Treatment regimen	Patient number (ESCC)	Median age	Male, *n* (%)	ECOG PS, *n* (%)		PD-L1 expression level		mOS (months)	mPFS (months)
						0	1	CPS, ≥10%	TPS, ≥1%		
RATIONALE 306 (2023)	Xu JM	Tislelizumab + TP/CF	326	64	282 (87)	109 (33)	217 (67)	116	NA	17.2	7.3
		Placebo + TP/CF	323	65	281 (87)	104 (32)	219 (68)	107	NA	10.6	5.6
KEYNOTE 590 (2021)	Sun JM	Pembrolizumab + CF	274^a^	64	306 (82)	149 (40)	223 (60)	143	NA	12.6	6.3
		Placebo + CF	274^a^	62	319 (85)	150 (40)	225 (60)	143	NA	9.8	5.8
CheckMate 648 (2022)	Doki Y	Nivolumab + CF	321	64	253 (79)	150 (47)	171 (53)	NA	158	13.2	5.8
		Placebo + CF	324	64	275 (85)	154 (48)	170 (52)	NA	157	10.7	5.6
ESCORT-1st (2021)	Luo H	Camrelizumab + TP	298	62	260 (87)	71 (23.8)	227 (76.2)	NA	166	15.3	6.9
		Placebo + TP	298	62	263 (88)	66 (22.1)	232 (77.9)	NA	163	12	5.6
ORIENT 15 (2022)	Lu Z	Sintilimab + TP/CF	327	63	279 (85)	77 (24)	250 (76)	188	NA	16.7	7.2
		Placebo + TP/CF	332	63	288 (87)	81 (24)	251 (76)	193	NA	12.5	5.7
JUPITER 06 (2022)	Wang ZX	Toripalimab + TP	257	63	217 (84)	66 (26)	191 (74)	NA	154	17.0	5.7
		Placebo + TP	257	62	220 (86)	68 (27)	189 (74)	NA	141	11.0	5.5
ASTRUM 007 (2023)	Song Y	Serplulimab + CF	368	64	317 (86)	93 (25)	275 (75)	162	NA	15.3	5.8
		Placebo + CF	183	64	153 (84)	53 (29)	130 (71)	79	NA	11.8	5.3

Abbreviation: PD-1, programmed death receptor 1; TP, paclitaxel plus cisplatin; CF, fluorouracil plus cisplatin; CPS, combined positive score; TPS, tumor proportion score; mOS, median overall survival; mPFS, median progression-free survival; ESCC, esophageal squamous cell carcinoma; ECOG PS, Eastern Cooperative Oncology Group Performance Status.

^a^ Only the ESCC, subgroup population from the KEYNOTE-590, trial was analyzed in this study.

A sum of seven Phase III trials were incorporated into the analysis. However, in KEYNOTE-590, the eligible patients had “unresectable or metastatic adenocarcinoma or squamous cell carcinoma of the esophagus or Siewert type 1 gastro-esophageal junction adenocarcinoma” (Sun et al., 2021). In this analysis, only the ESCC subgroup was included, and the characteristics of the ESCC subgroup were not reported in the original. Moreover, the arm with nivolumab plus ipilimumab in CheckMate 648 was not included. A total of 4,162 participants with previously untreated, histologically or cytologically confirmed, locally advanced, unresectable, or metastatic ESCC were included. All of the subjects were randomly assigned to receive first-line treatment, with 2,171 patients receiving PD-1 inhibitors plus platinum-based chemotherapy doublet and 1,991 patients receiving chemotherapy alone.

**FIGURE 2:**
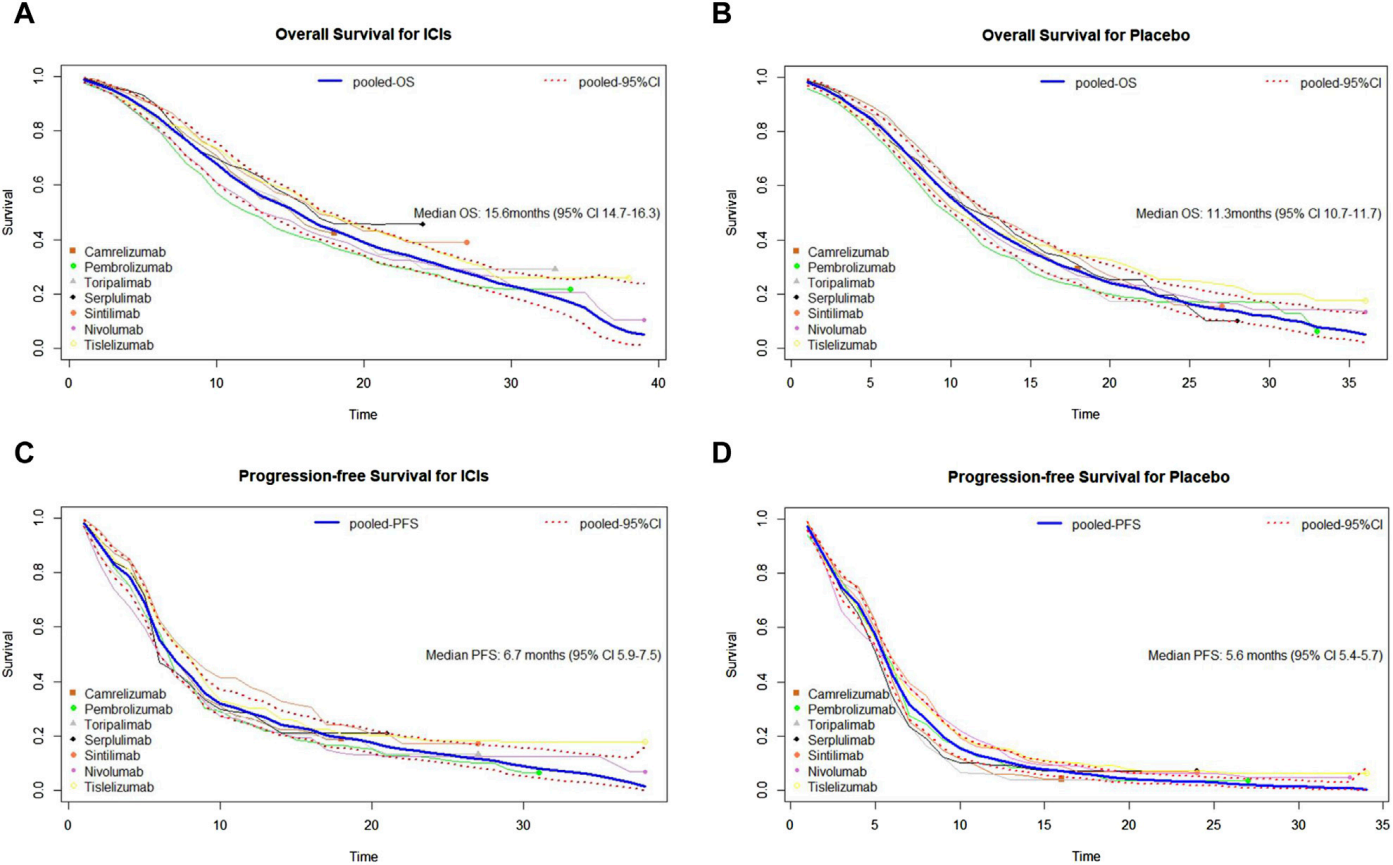
Pooled curves of the overall survival and progression-free survival in the seven studies. **(A)** Pooled overall survival (OS) Kaplan–Meier for ICIs plus chemotherapy. **(B)** Pooled OS Kaplan–Meier for placebo plus chemotherapy. **(C)** Pooled progression-free survival (PFS) Kaplan–Meier for immune checkpoint inhibitors (ICIs) plus chemotherapy. **(D)** Pooled PFS Kaplan–Meier for placebo plus chemotherapy. The bold blue lines represent the summarized survival curves with the 95% confidence bands (dashed lines) obtained using our approach MetaSurv. ICIs, immune checkpoint inhibitors; OS, overall survival; PFS, progression-free survival.

To evaluate the survival efficacy of immunotherapy-based regimens in comparison with chemotherapy alone in the first-line treatment of patients with advanced ESCC, we pooled the survival curves from seven clinical trial groups (Figure 2). Mild heterogeneity was noted in the combination of PFS curves of PD-1 agents based on *I* ^2^ = 8.75% and the lower limit of *H* value confidence interval (*LL*) = 1.04 (Supplementary Table S1). Thus, a random-effect model was used. However, there was no heterogeneity when merging other survival curves depending on *I* ^2^ = 0 and *LL* < 1 (Supplementary Table S1). We used fixed-effect models to merge the curves. Moreover, detailed subgroup analyses of basic characteristics were conducted for OS and PFS, using six variables. The results showed that sex, age, race, ECOG PS, disease status at trial entry, and chemotherapy did not impact the heterogeneity between groups, with all *p*-values greater than 0.05 (Supplementary Table S2). Thus, the results showed that patients from seven trials were comparable. In this study, the median OS of ICIs plus chemotherapy group after synthesis was 15.6 months (95% CI: 14.7–16.3), and the median PFS was 6.7 months (95% CI: 5.9–7.5). As for the chemotherapy group, the synthetic median OS was 11.3 months (95% CI: 10.7–11.7), and the median PFS was 5.6 months (95% CI: 5.4–5.7). Significantly, there was a risk reduction of death in the ICI group when compared with chemotherapy.

**FIGURE 3:**
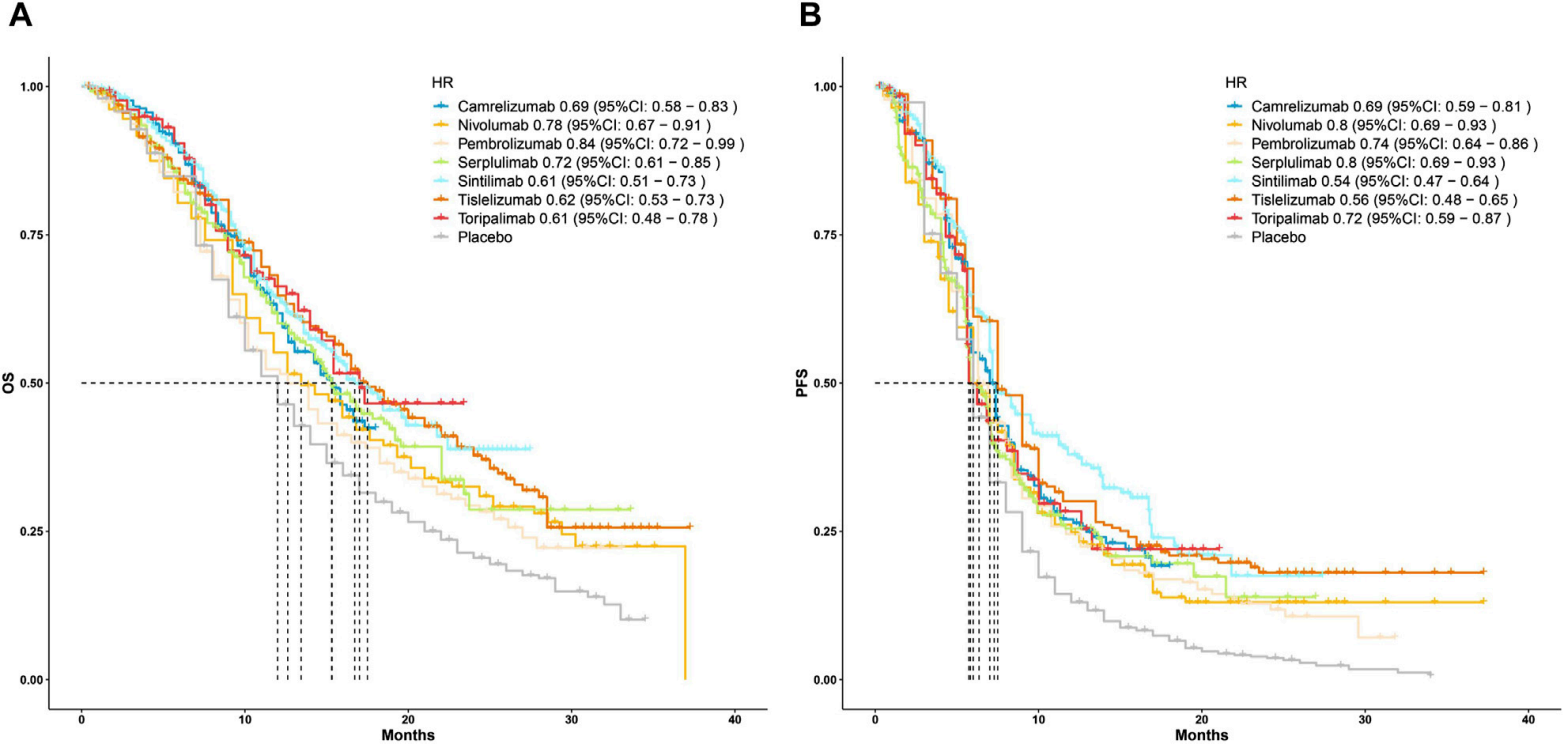
Kaplan–Meier curves for **(A)** overall survival and **(B)** progression-free survival that compared PD-1 inhibitor plus chemotherapy with pooled placebo plus chemotherapy. HR, hazard ratio; OS, overall survival; PFS, progression-free survival.

**FIGURE 4:**
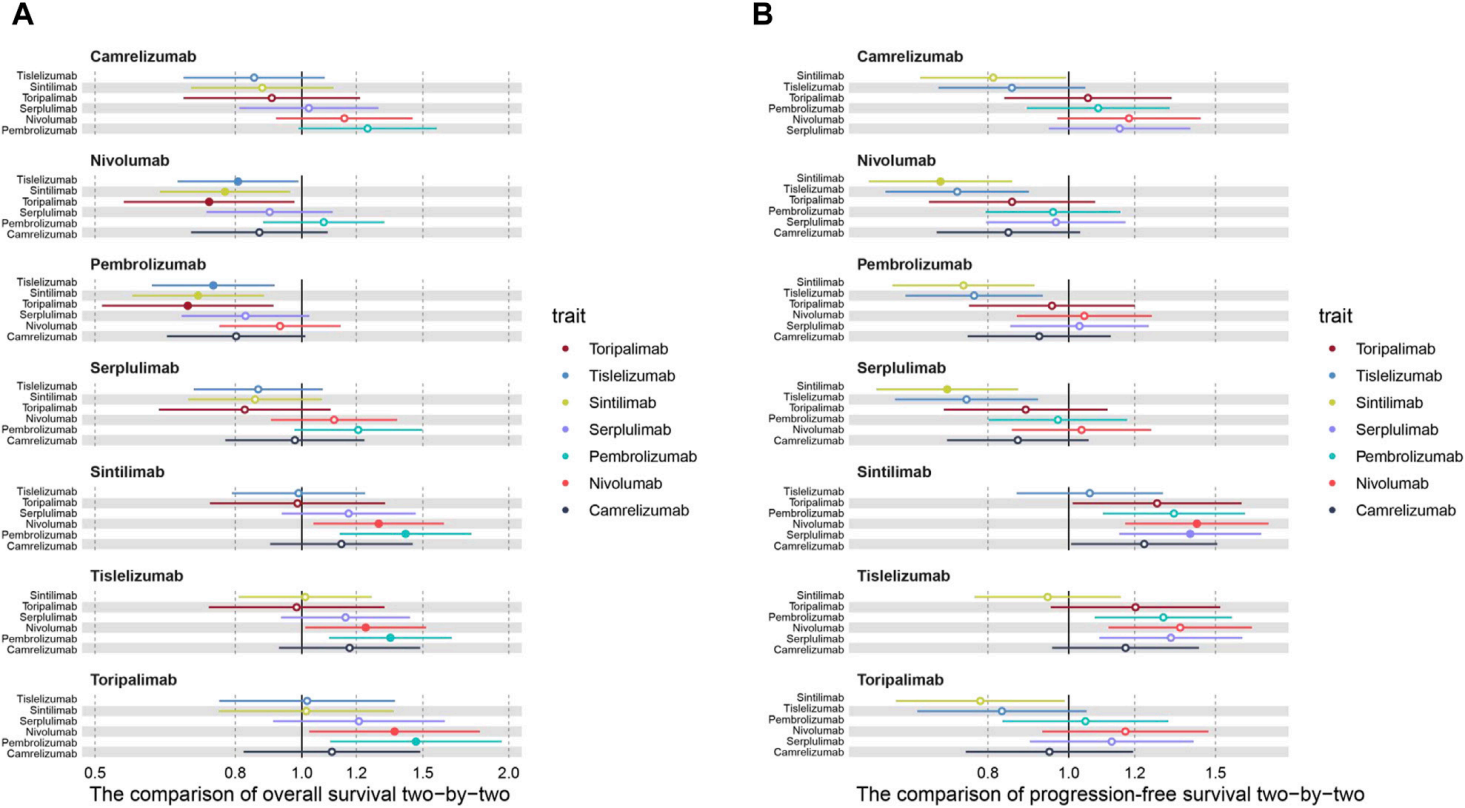
Hazard ratios of the reconstructed **(A)** overall survival and **(B)** progression-free survival comparisons two by two.

Subsequently, to compare the survival benefits between the seven regimens, we reconstructed the individual patient data (IPD) of seven trials. Figure 3 shows the OS and PFS curves of seven PD-1 inhibitors plus chemotherapy and the pooled placebo group. Moreover, survival analyses were conducted two by two based on the reconstructed IPD. As shown in Figure 4A, the toripalimab, tislelizumab, and sintilimab group had the best OS performance among the seven ICI groups (median OS: 17.0 months, 17.2 months, and 16.7 months, Supplementary Figure S1). Furthermore, the pembrolizumab and nivolumab group showed unsatisfied OS benefit (median OS: 12.6 months and 13.2 months, Supplementary Figure S1). For PFS, sintilimab and tislelizumab were superior to other regimens (Figure 4B) with a median PFS of 7.2 and 7.3 months, respectively (Supplementary Figure S2).

**FIGURE 5:**
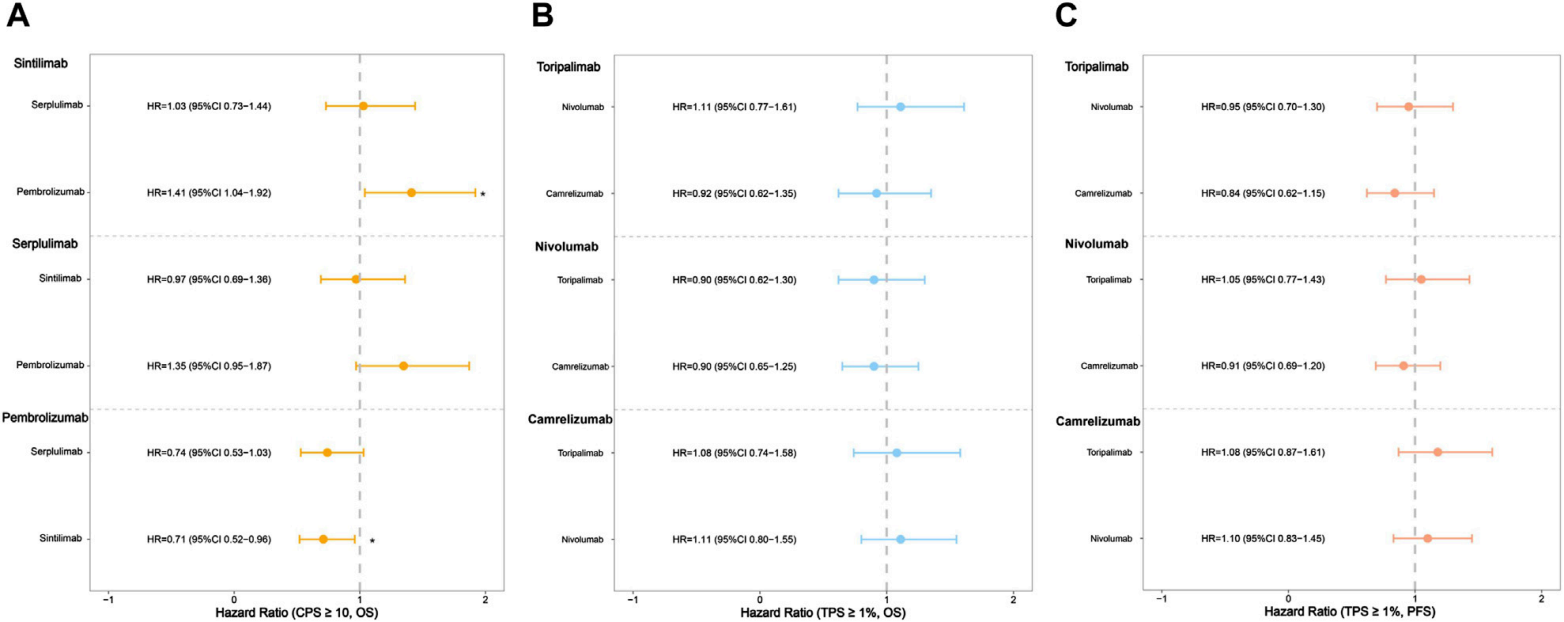
Hazard ratios of the reconstructed overall survival (OS) and progression-free survival (PFS) comparisons in subgroups two by two (^*^ *p* < 0.05). **(A)** The comparison of OS in subgroups of patients with CPS ≥10. **(B)** The comparison of OS in subgroups of patients with TPS ≥1%. **(C)** The comparison of PFS in subgroups of patients with TPS ≥1%.

Additionally, we conducted subgroup analyses with respect to PD-L1 status. Three of the seven trials reported OS curves in subgroups of patients with CPS ≥10. Sintilimab showed significant OS benefits in the cohort of high PD-L1 expression level, in comparison with pembrolizumab (HR: 0.71, 95% CI: 0.52–0.96; Figure 5A; Supplementary Figure S3). Serplulimab showed similar efficacy to sintilimab (HR: 1.03, 95% CI: 0.73–1.44; Figure 5A; Supplementary Figure S3) in the patients with a high PD-L1 expression level, due to the 95% CI of HR containing 1, which showed no significant differences. In terms of the TPS ≥1% group, three regimens (camrelizumab, nivolumab, and toripalimab) performed similar survival efficacy in both OS and PFS (Figures 5B,C; Supplementary Figure S4-S5) because all of the 95% CIs of HR contained 1.

To further verify the heterogeneity, we merged HR values published in seven trials (Supplementary Figure S6). There was no heterogeneity among the OS outcomes in the seven trials (*I* ^2^ = 0.00%, *p* = 0.87). As for PFS, there was a mild heterogeneity (*I* ^2^ = 18.30%, *p* = 0.29), which was in keeping with the merging of PFS curves.

Risk of bias was assessed using the Cochrane risk of bias assessment tool. Details of the bias risk assessment for each study are summarized in Supplementary Figure S7. High risk of bias was mainly caused by the selection of reported results. Lastly, sensitivity analyses were performed to evaluate the robustness of the merging. The pooled raw HRs for OS and PFS were stable, indicating that individual research had no discernible impact on the pooled results (Supplementary Figure S8).

## 4 Discussion

Patients with advanced ESCC commonly have a poor quality of life, resulting from neoplasm invasiveness. National Comprehensive Cancer Network and European Clinical Guidelines both preferred fluoropyrimidine and cisplatinum as the first-line chemotherapy regimen (Ajani et al., 2013; Waddell et al., 2014). In Asia, paclitaxel plus platinum is the mainstream treatment. With the progress of new medical research, immunotherapy has demonstrated superior efficacy over standard chemotherapy in ESCC. Currently, a sum of trials of PD-1 inhibitors combined with platinum-based chemotherapy in ESCC have met their primary endpoints. The striking therapeutic effects propelled PD-1 inhibitors cored regimen from a later treatline to the first line in more treatment guidelines of ESCC.

In this analysis, we evaluated the survival efficacy of the currently recommended PD-1 inhibitor–based regimens in the first line of advanced ESCC. A comparison of survival efficacy of most network meta-analyses was conducted using the HR value from clinical trials, which may just generate the ranking status of drugs but may be hard to reflect the global survival differences among drugs. Our analysis based on reconstructed IPD further displayed a detailed comparison of the survival efficacies among various regiments at different time points. Conversely, merging the survival curves of a specific population can fully reflect the survival situation of a general specific population by expanding the sample size. Thus, we combined chemotherapy groups from different trials to provide a comprehensive performance of the control group in our study. In this study, we merged survival curves of seven trials to reflect the antitumor activity of PD-1-based regimens compared with chemotherapy alone in patients with advanced ESCC. Moreover, we employed the individual reconstruction IPD method to make two-by-two comparisons among different groups of PD-1 plus chemotherapy, which directly reflected the detailed survival outcomes of different regimens. Overall, we believe that this approach may be a good complement to traditional HR analysis, and it helps to manage the survival data from a more holistic perspective.

By synthesizing the OS and PFS curves of seven PD-1 inhibitors combined with chemotherapy or chemotherapy alone, we found that PD-1 inhibitors plus chemotherapy generated significant benefits of OS over chemotherapy alone. Although the pooled median PFS was relatively close, the 1-year PFS rates of the PD-1 group were far higher than chemotherapy group (Wang et al., 2022; Song et al., 2023), which may be due to the heterogeneity of immunotherapy response (Galon and Bruni, 2019). In two-by-two comparisons, tislelizumab, sintilimab, and toripalimab combined with chemotherapy showed the best OS benefit, whereas pembrolizumab plus chemotherapy presented the shortest OS than other treatments. As for PFS, the sintilimab and tislelizumab group displayed lower recurrent risk than other regimens, which may contribute to reducing the financial burden by delaying the progression and recurrence of the disease.

Nowadays, potential predictors for prognosis and immunotherapy responses have been extensively explored. PD-L1 expression has been widely employed as a valuable biomarker in recent trials of varieties of malignancies (Topalian et al., 2016). We separately compared the survival efficacies of PD-1 inhibitors based on two scoring algorithms (TPS and CPS). Subgroup analysis showed that the death risk of the pembrolizumab group was significantly higher than the other treatments, which was inconsistent with results from previous studies (Liang et al., 2020; Xu et al., 2023). Therefore, ongoing research is required to explain this discrepancy.

Different chemotherapy schemes may have different antitumor efficacies. In a meta-analysis by Zhao J., PD-1 inhibitors with paclitaxel/cisplatinum were superior to fluoropyrimidine/cisplatinum (Zhao et al., 2023). Several studies reported that taxane induces immunogenic cell death of cancer cells and causes various immunogenic actions, which creates favorable conditions to enhance the immune response of PD-1 inhibitors (Chan and Yang, 2000; Garnett et al., 2008; Galluzzi et al., 2020). KEYNOTE-590, CheckMate 648, and ASTRUM-007 used fluoropyrimidine/cisplatinum as the chemotherapy scheme, which may result in a poorer survival outcome to some extent. There remains an urgent need for high-quality studies of comparison among different chemotherapy regimens.

## 5 Limitations

There were still some limitations in our survival analysis method. Due to the unavailable head-to-head clinical trials, these results still need to be treated dialectically. The mild heterogeneities in the included studies may be caused by smoking status, alcohol use, and other baseline differences. However, a subgroup analysis considering these covariates is not yet possible because of the lack of survival outcomes. Future analysis is warranted after more treatment results are reported. Second, concerning the reconstruction of the deleted data, the occurrence rate of the deleted cases was assumed to be fixed in a single time interval. However, this assumption was based on that the occurrence of the deleted cases is not affected by time-related confounding factors, which may be different from the actual situation in clinical trials. Third, more studies must be enrolled in future analyses, and the IPDfromKM method should be combined with the Bayesian network analysis method. Additionally, because of the inconsistency of PD-L1 expression algorithm methods, the subgroup analysis of PD-L1 status was not comprehensive. Finally, owing to the manageable toxicity and low incidence rate, adverse events caused by immunotherapy were not analyzed in this study.

## 6 Conclusion

In conclusion, PD-1 inhibitors combined with chemotherapy significantly enhanced the OS time of patients with advanced ESCC. Toripalimab, tislelizumab, and sintilimab plus chemotherapy performed the best OS benefit. Patients of sintilimab and tislelizumab had a relatively low risk of disease recurrence and metastasis. For patients with ESCC with high PD-L1 abundance, sintilimab was strongly recommended. To provide more guidance to support healthcare decision-making and precision medicine, further real-world comparative studies are still needed.

## Data Availability

The original contributions presented in the study are included in the article/Supplementary Material; further inquiries can be directed to the corresponding author.
