# Supplementary material for: PD-1 inhibitors in advanced esophageal squamous cell carcinoma: a survival analysis of reconstructed patient-level data
Source: Front Pharmacol. 2024 Jul 18;15:1408458. doi: 10.3389/fphar.2024.1408458 (PMC11291229; doi:10.3389/fphar.2024.1408458)
Supplement: Supplementary file 1 [file DataSheet1.docx]

Supplementary Material

**Supplementary Figure S1.** Reconstructed median OS comparisons between PD-1 inhibitors.

**Supplementary Figure S2.** Reconstructed median PFS comparisons between PD-1 inhibitors.

**Supplementary Figure S3.** Reconstructed Kaplan-Meier plot of overall survival comparisons between PD-1 inhibitors in patients with PD-L1 CPS≥10.

**Supplementary Figure S4.** Reconstructed Kaplan-Meier plot of overall survival comparisons between PD-1 inhibitors in patients with PD-L1 TPS≥1%.

**Supplementary Figure S5.** Reconstructed Kaplan-Meier plot of progression-free survival comparisons between PD-1 inhibitors in patients with PD-L1 TPS≥1%.

**Supplementary Figure S6.** Forest plot for (A) overall survival and (B) progression-free survival that compared PD-1 inhibitor plus chemotherapy with chemotherapy alone in esophageal cancer patients.

**Supplementary Figure S7.** Details of the bias risk assessment for each study.

**Supplementary Figure S8.** Results of sensitivity analyses by excluding of the listed trials.

**Supplementary** **Table S1.** Heterogeneity analysis in the meta-analysis of survival after synthesis.

**Supplementary Table S2.** Subgroup analysis of overall survival and progression-free survival.

| (A)  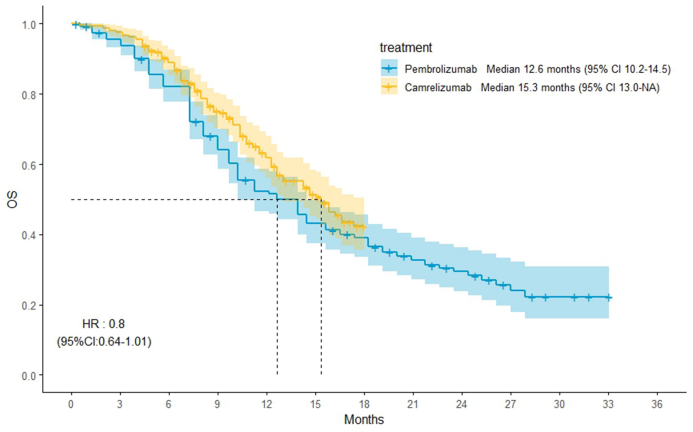 | (B)  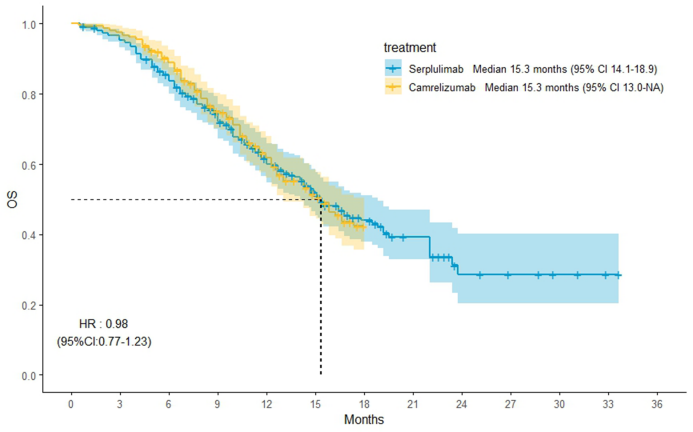 |
| --- | --- |
| (C)  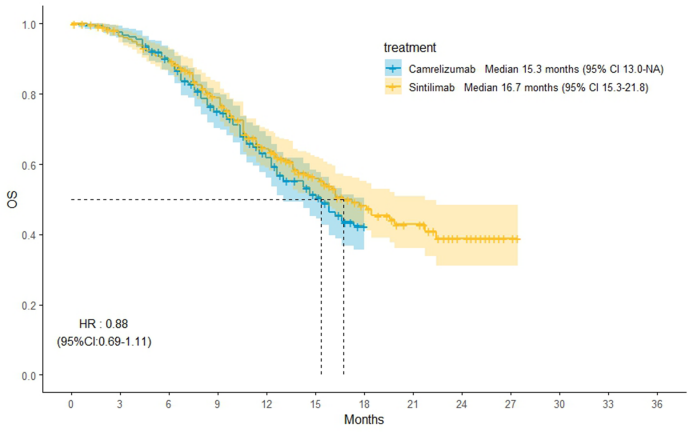 | (D)  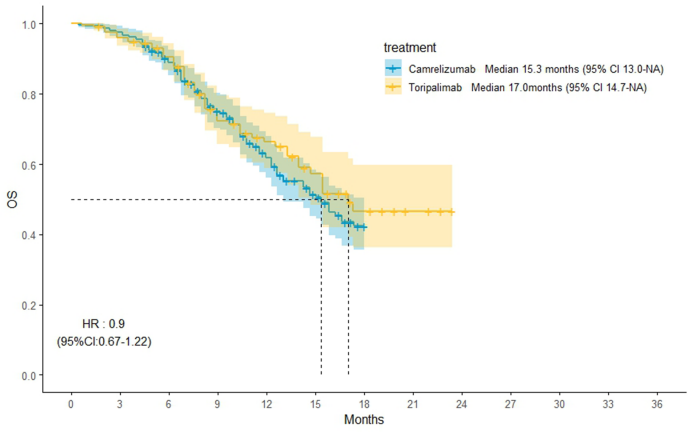 |
| (E)  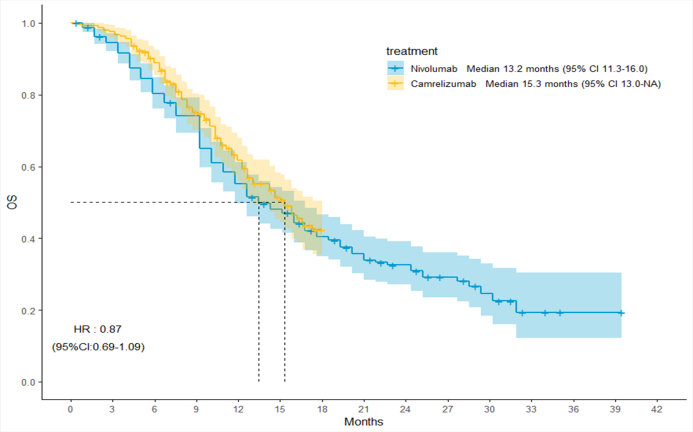 | (F)  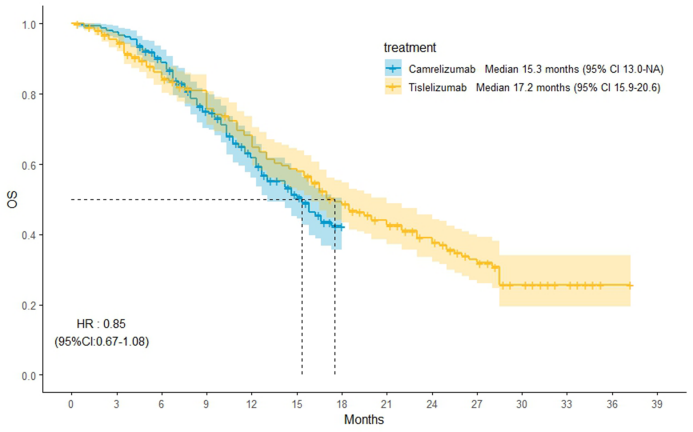 |
| (G)  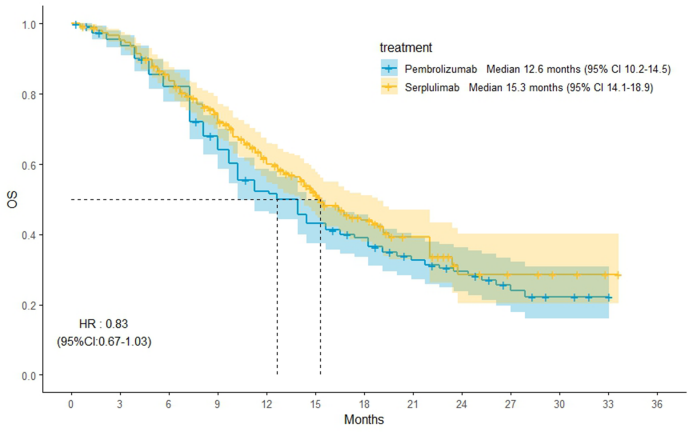 | (H)  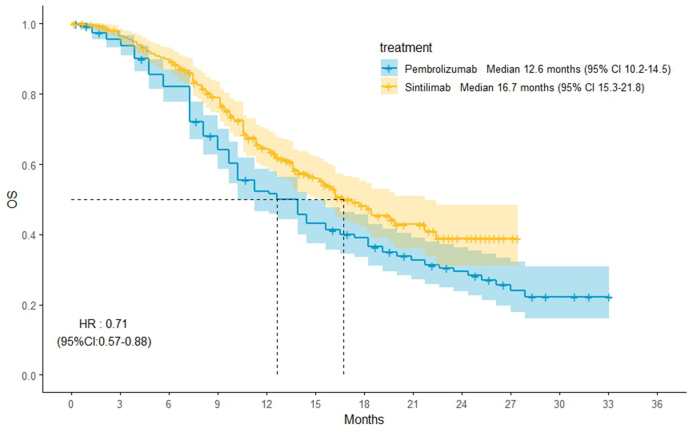 |
| (I)  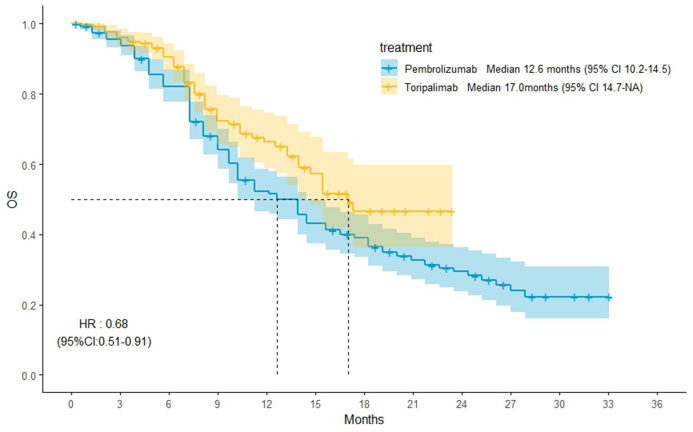 | (J)  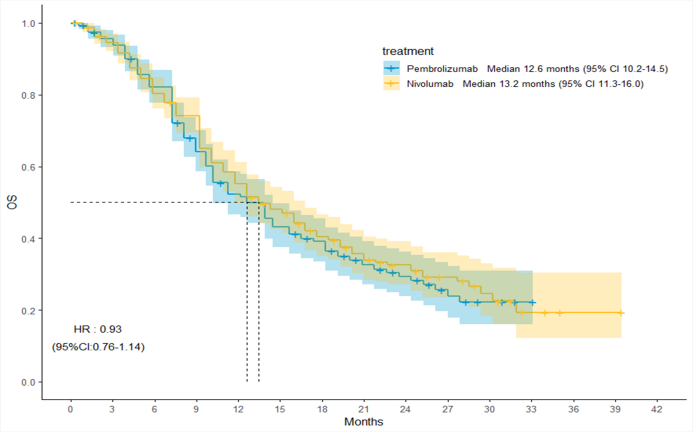 |
| (K)  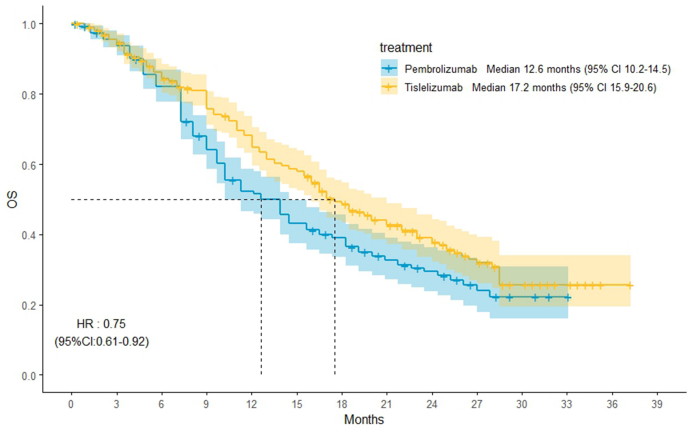 | (L)  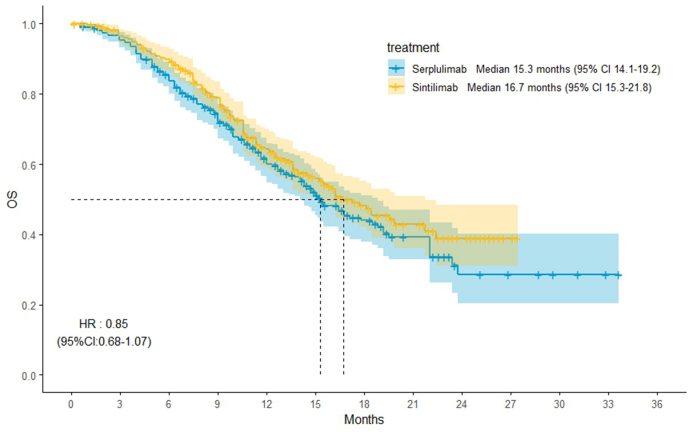 |
| (M)  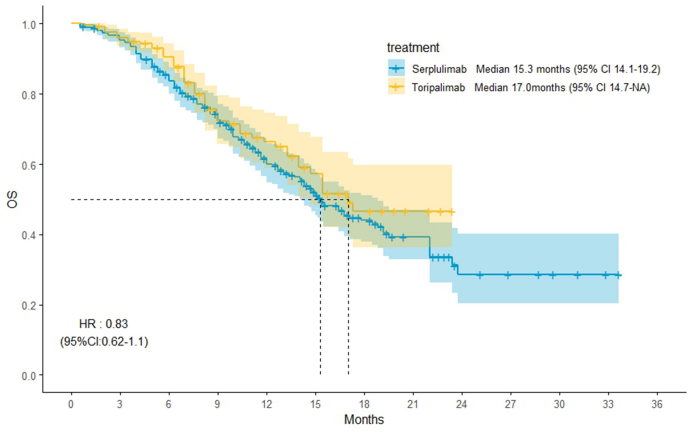 | (N)  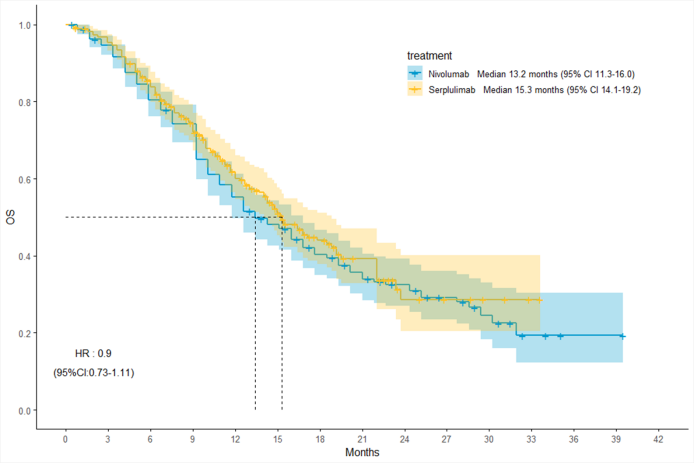 |
| (O)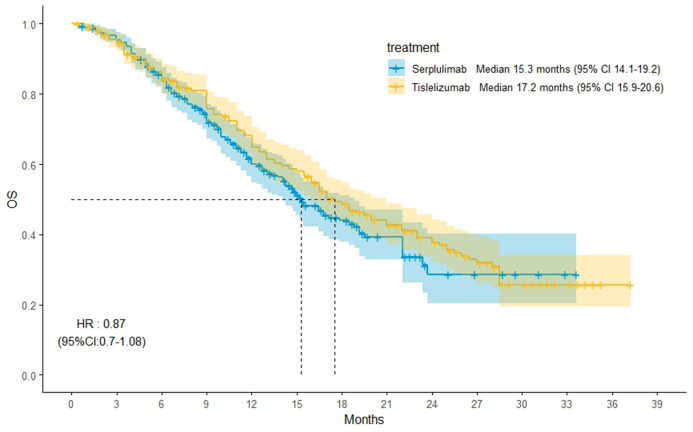 | (P)  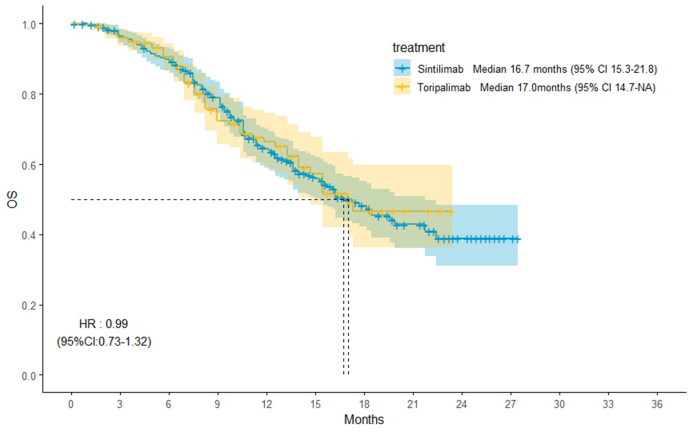 |
| (Q)  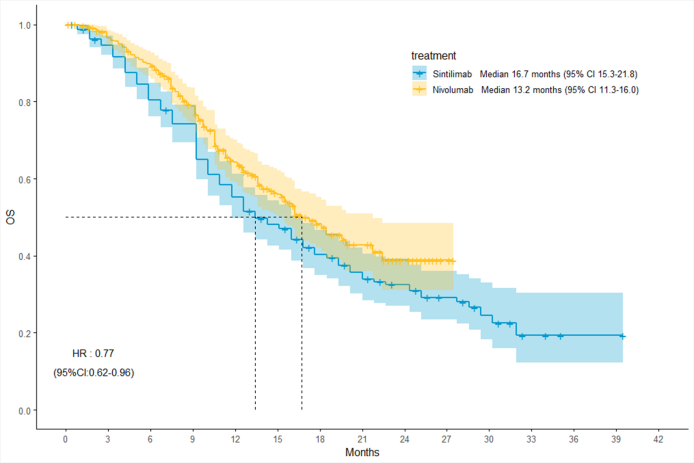 | (R)  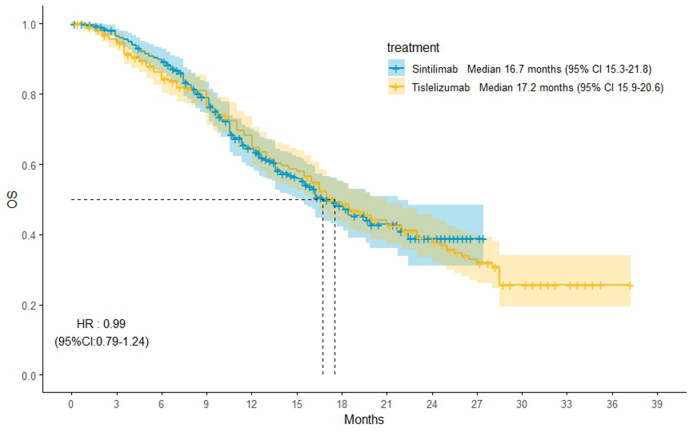 |
| (S)  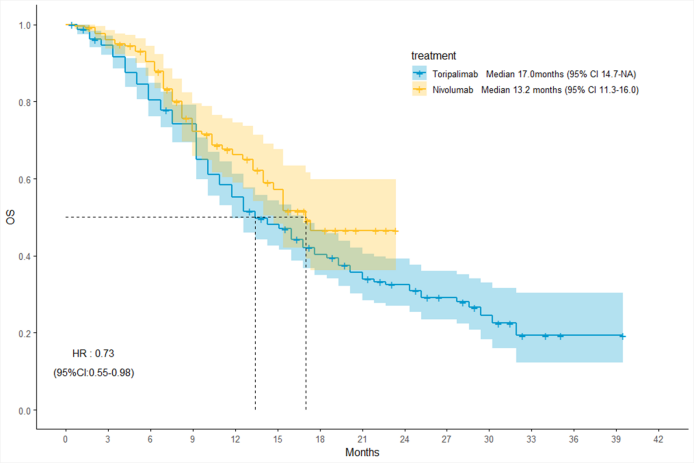 | (T)  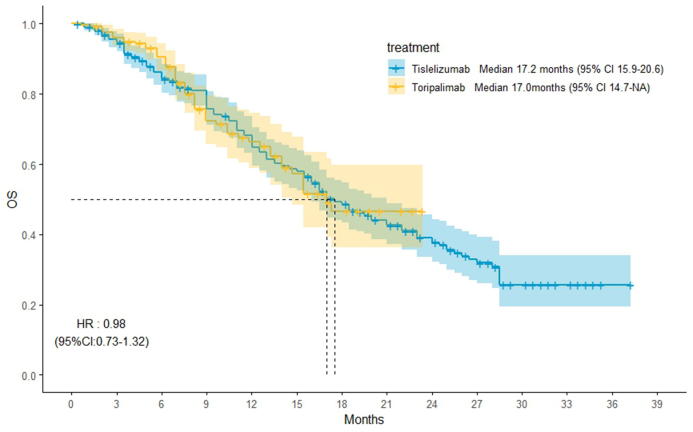 |
| (U)  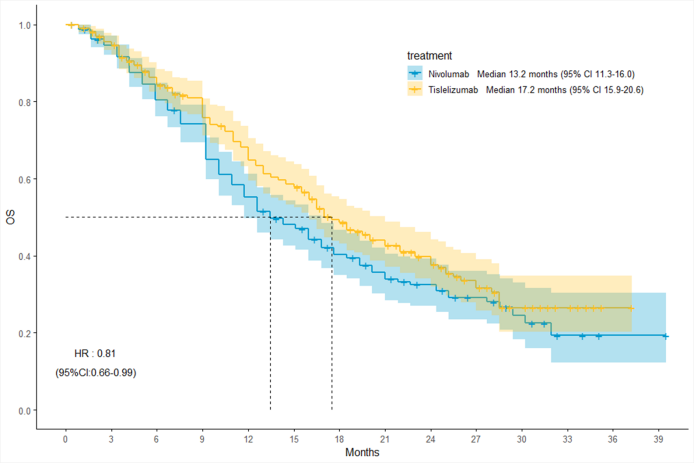 |  |

**Supplementary Figure S1.** Reconstructed Kaplan-Meier plot of overall survival comparisons between PD-1 inhibitors in the ITT population.

1. Camrelizumab vs. Pembrolizumab; (B) Camrelizumab vs. Serplulimab; (C) Camrelizumab vs. Sintilimab; (D) Camrelizumab vs. Toripalimab; (E) Camrelizumab vs. Nivolumab; (F) Camrelizumab vs. Tislelizumab; (G) Pembrolizumab vs. Serplulimab; (H) Pembrolizumab vs. Sintilimab; (I) Pembrolizumab vs. Toripalimab; (J) Pembrolizumab vs. Nivolumab; (K) Pembrolizumab vs. Tislelizumab; (L) Serplulimab vs. Sintilimab; (M) Serplulimab vs. Toripalimab; (N) Serplulimab vs. Nivolumab; (O) Serplulimab vs. Tislelizumab; (P) Sintilimab vs. Toripalimab; (Q) Sintilimab vs. Nivolumab; (R) Sintilimab vs. Tislelizumab; (S) Toripalimab vs. Nivolumab; (T) Toripalimab vs. Tislelizumab; (U) Nivolumab vs. Tislelizumab.

| (A)  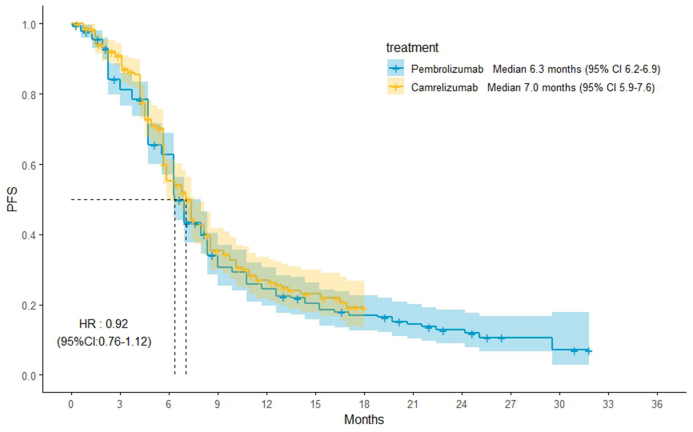 | (B)  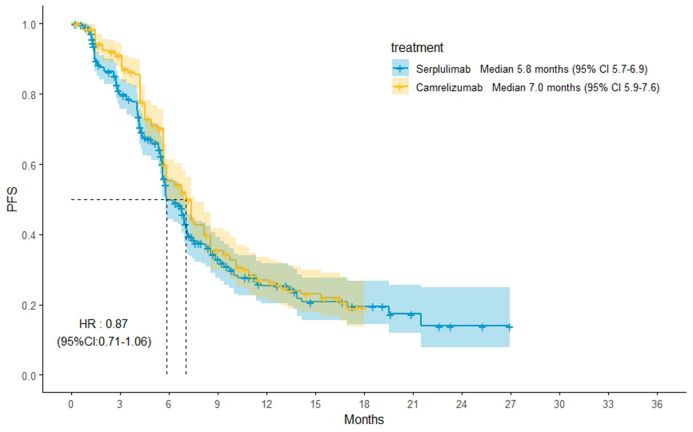 |
| --- | --- |
| (C)  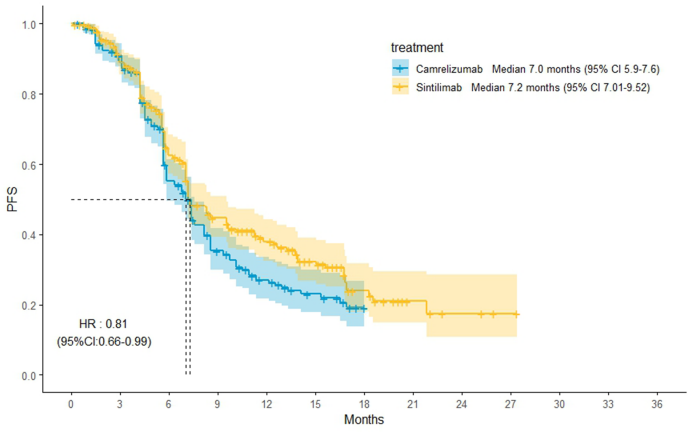 | (D)  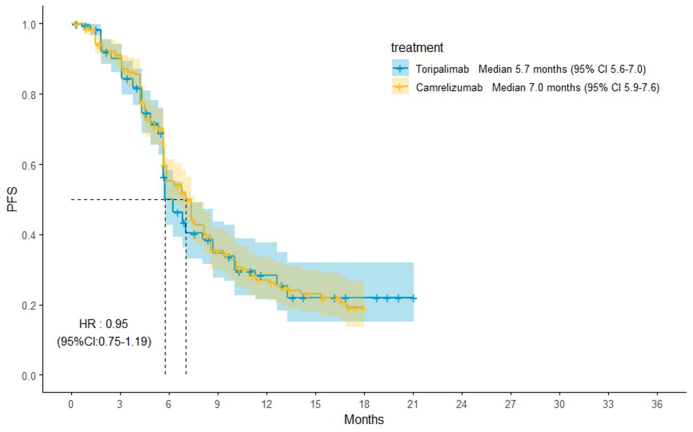 |
| (E)  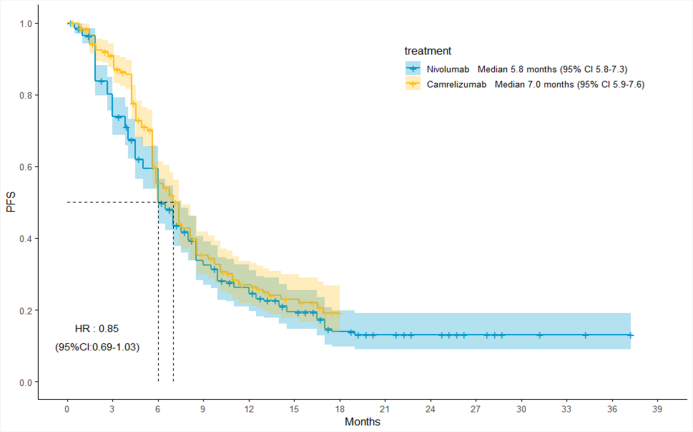 | (F)  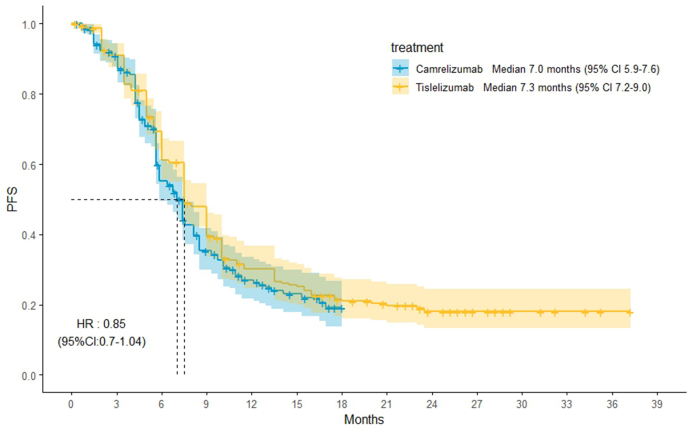 |
| (G)  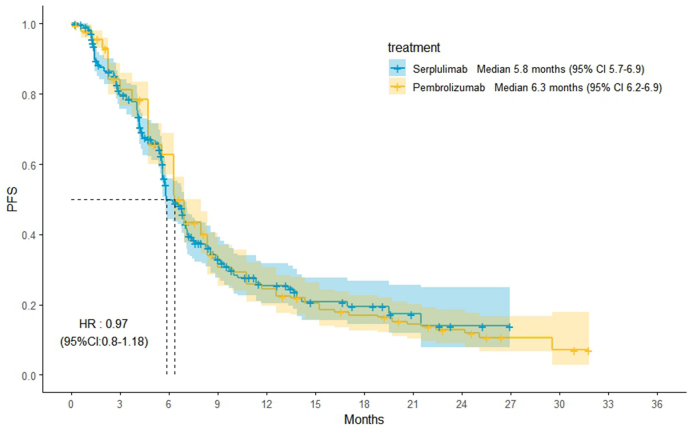 | (H)  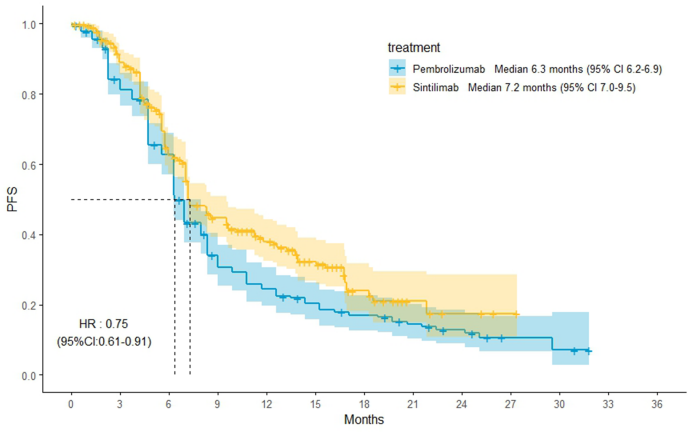 |
| (I)  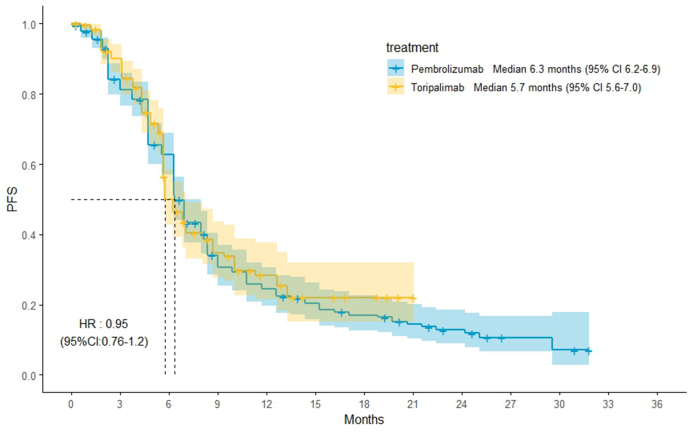 | (J)  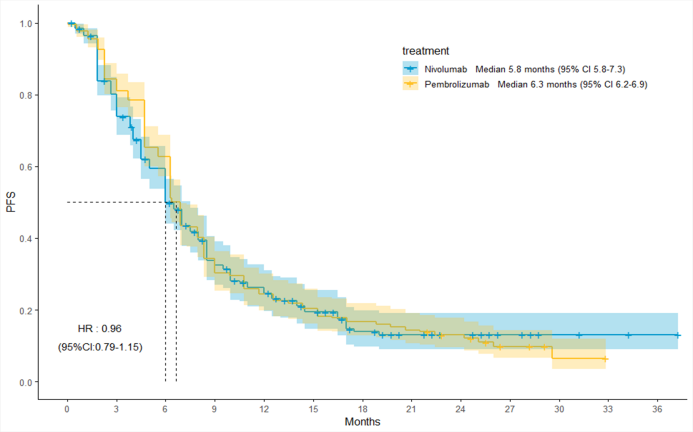 |
| (K)  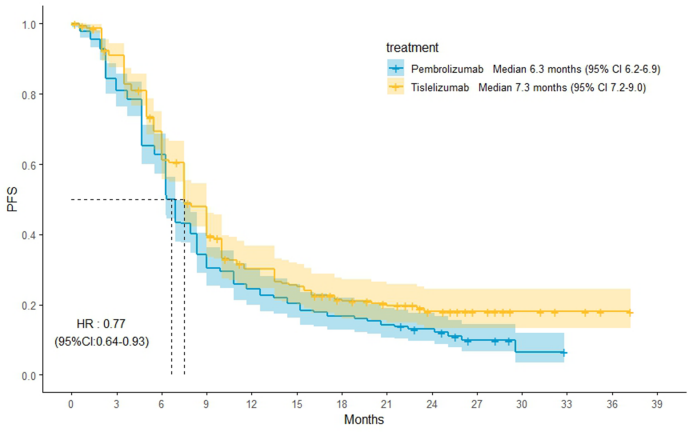 | (L)  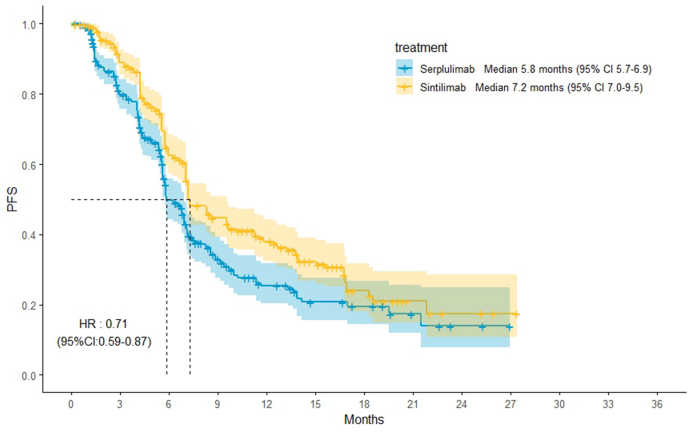 |
| (M)  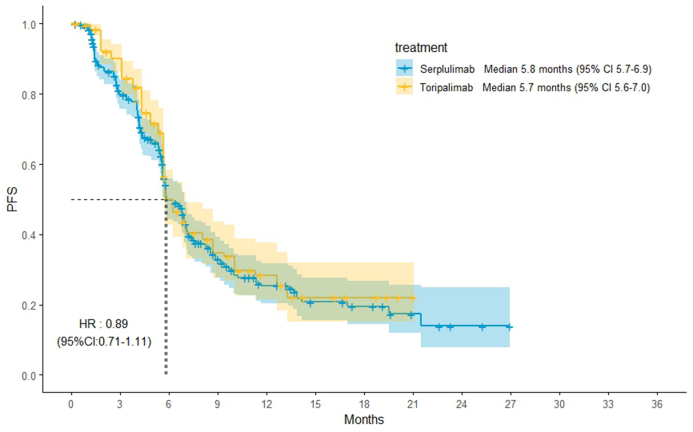 | (N)  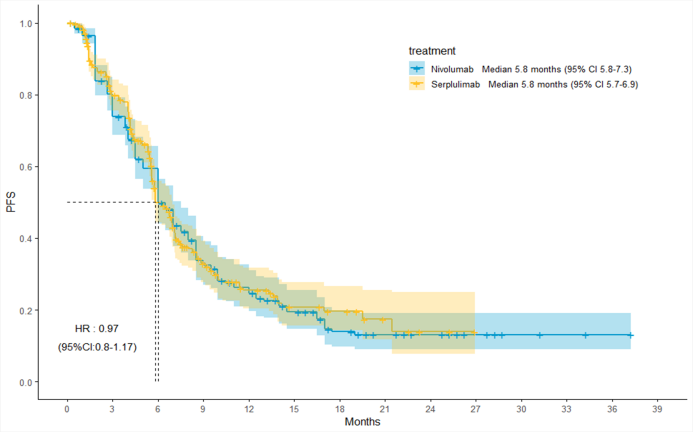 |
| (O)  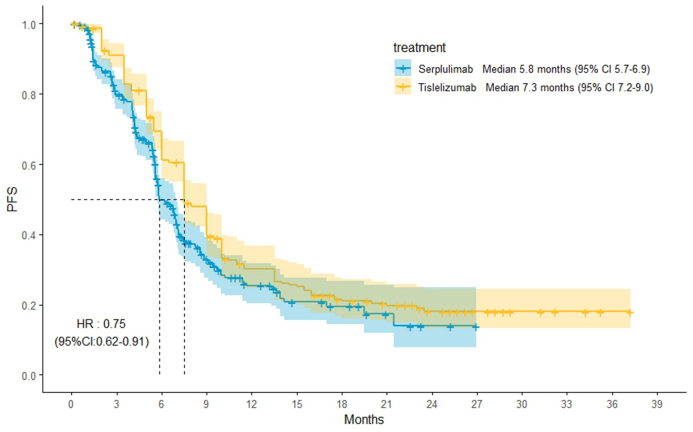 | (P)  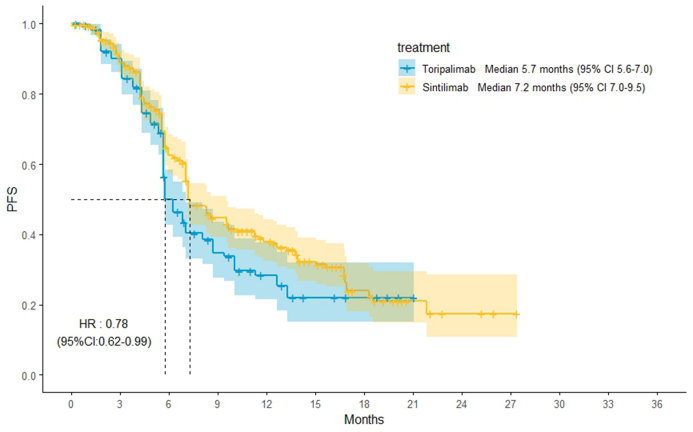 |
| (Q)  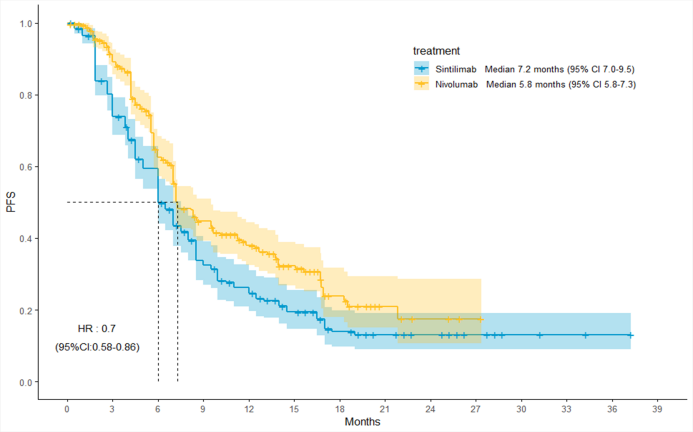 | (R)  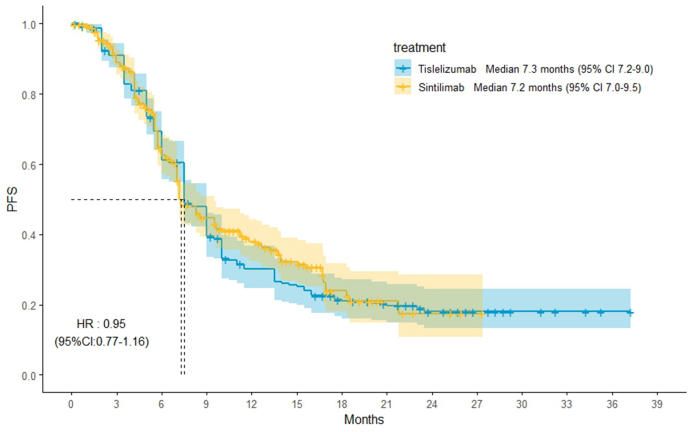 |
| (S)  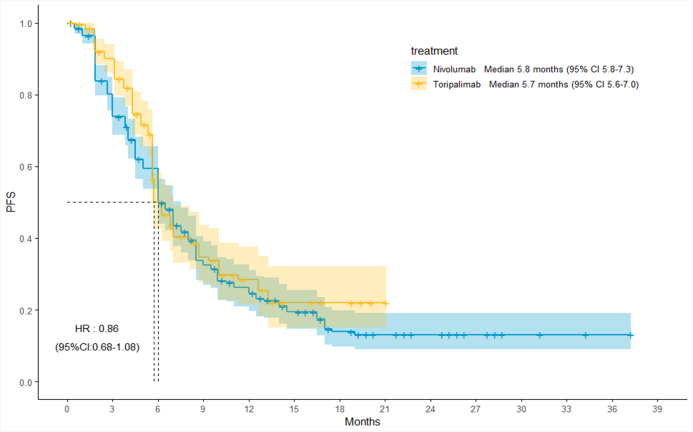 | (T)  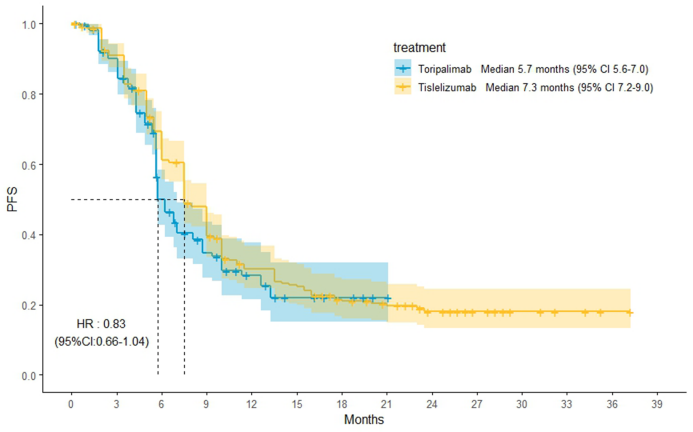 |
| (U)  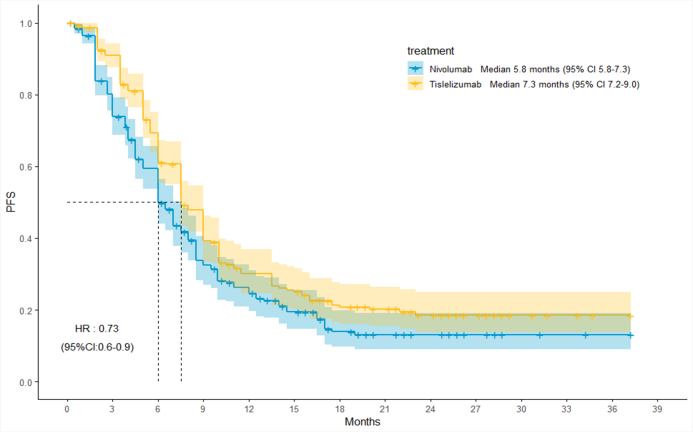 |  |

**Supplementary Figure S2.** Reconstructed Kaplan-Meier plot of progression-free survival comparisons between PD-1 inhibitors in the ITT population.

1. Camrelizumab vs. Pembrolizumab; (B) Camrelizumab vs. Serplulimab; (C) Camrelizumab vs. Sintilimab; (D) Camrelizumab vs. Toripalimab; (E) Camrelizumab vs. Nivolumab; (F) Camrelizumab vs. Tislelizumab; (G) Pembrolizumab vs. Serplulimab; (H) Pembrolizumab vs. Sintilimab; (I) Pembrolizumab vs. Toripalimab; (J) Pembrolizumab vs. Nivolumab; (K) Pembrolizumab vs. Tislelizumab; (L) Serplulimab vs. Sintilimab; (M) Serplulimab vs. Toripalimab; (N) Serplulimab vs. Nivolumab; (O) Serplulimab vs. Tislelizumab; (P) Sintilimab vs. Toripalimab; (Q) Sintilimab vs. Nivolumab; (R) Sintilimab vs. Tislelizumab; (S) Toripalimab vs. Nivolumab; (T) Toripalimab vs. Tislelizumab; (U) Nivolumab vs. Tislelizumab.

| (A)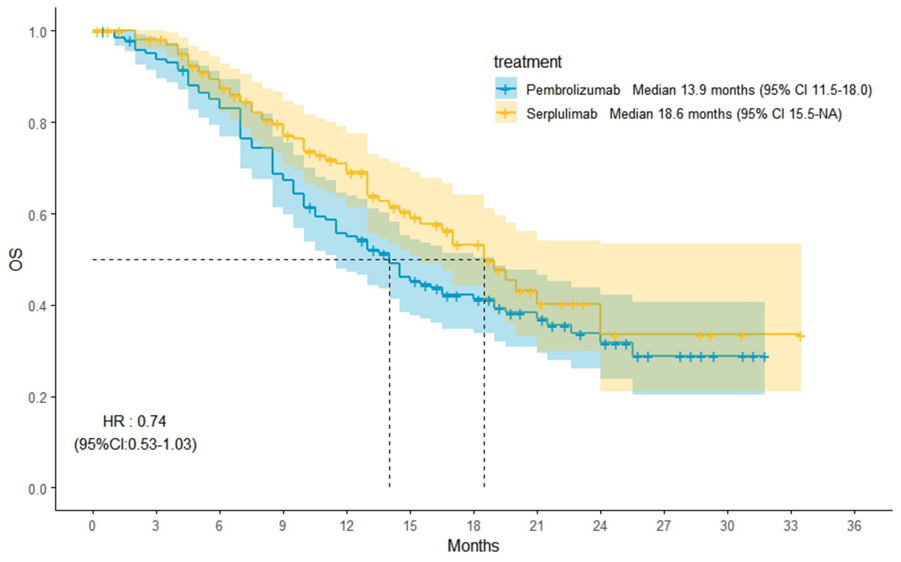 |
| --- |
| (B)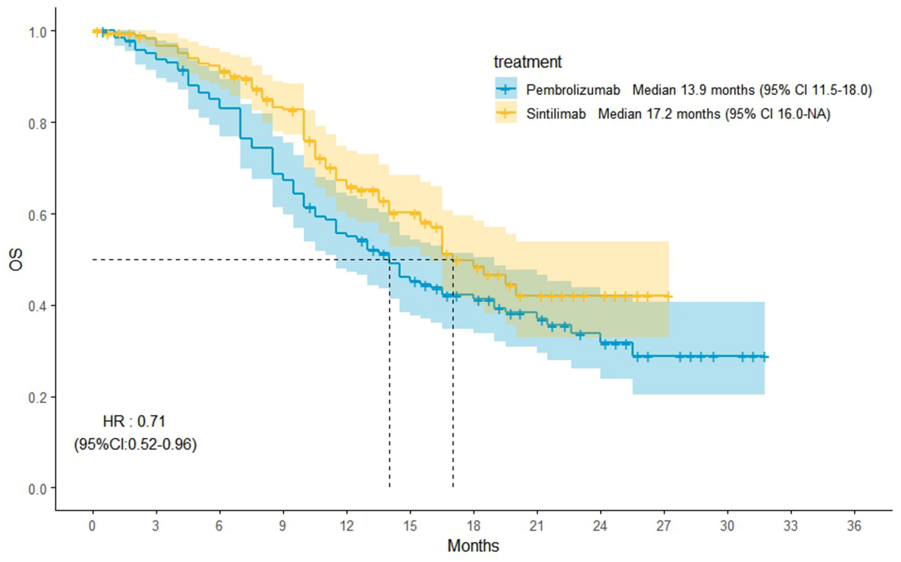 |
| (C)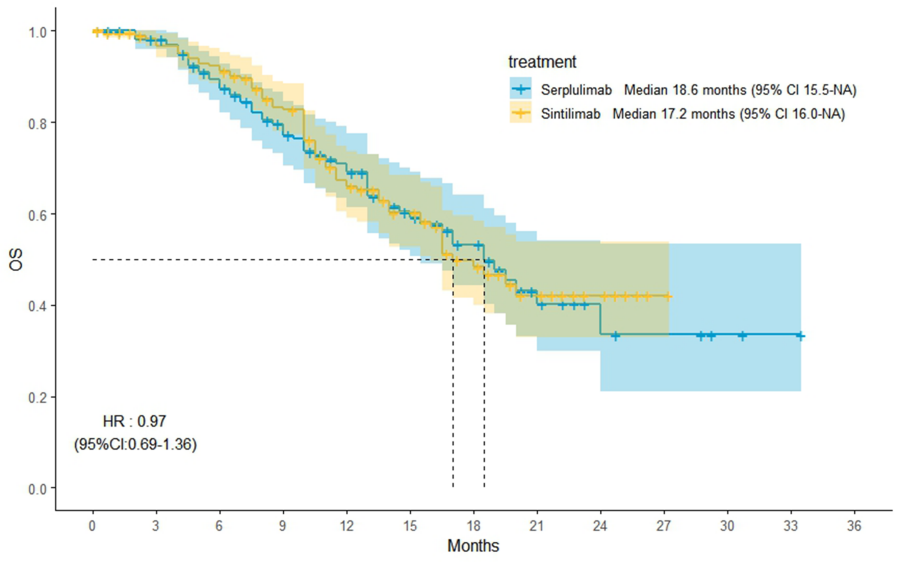 |

**Supplementary Figure S3.** Reconstructed Kaplan-Meier plot of overall survival comparisons between PD-1 inhibitors in patients with PD-L1 CPS≥10.

1. Pembrolizumab vs. Serplulimab; (B) Pembrolizumab vs. Sintilimab; (C) Serplulimab vs. Sintilimab.

| (A)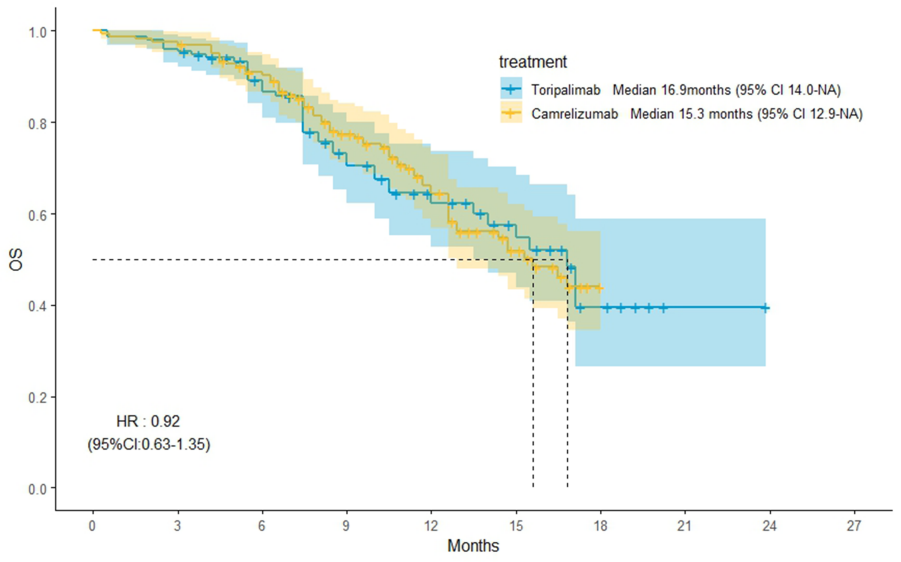 |
| --- |
| (B)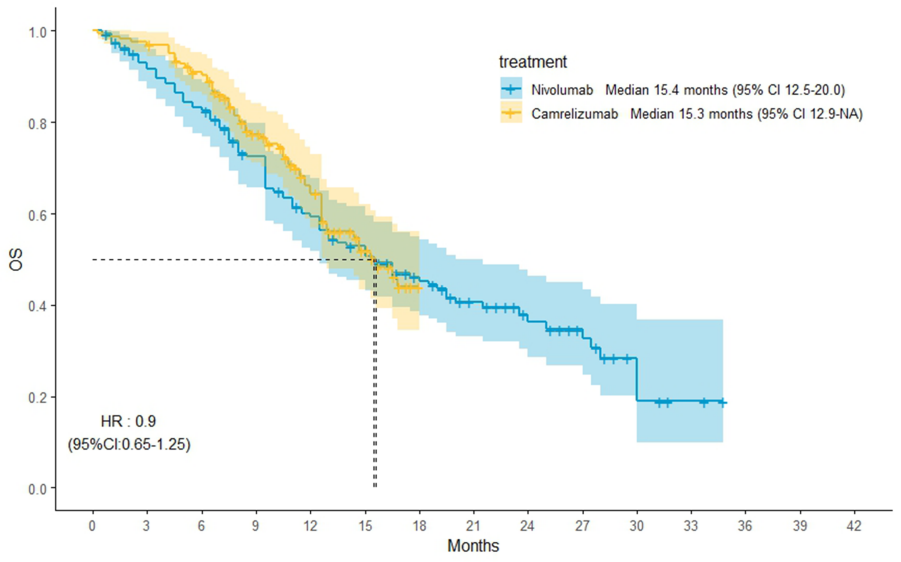 |
| (C)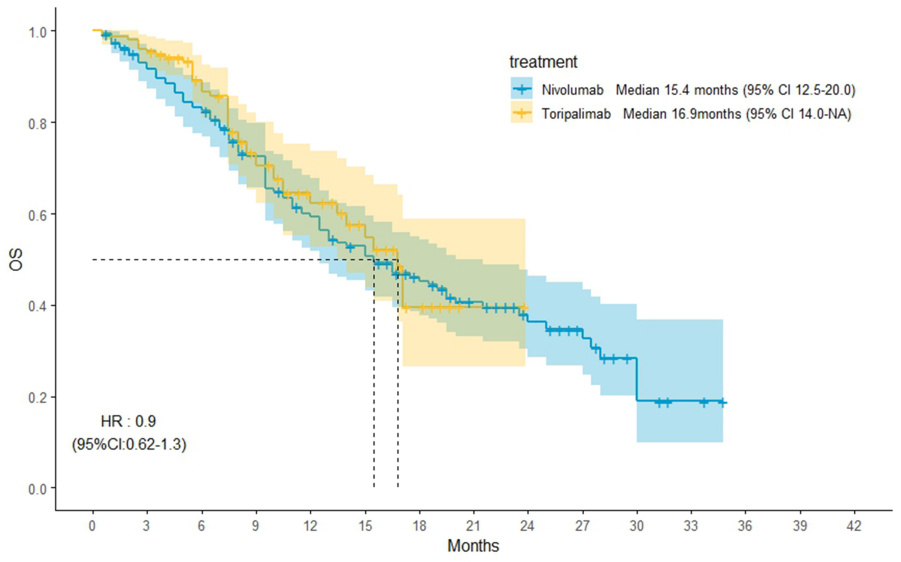 |

**Supplementary Figure S4.** Reconstructed Kaplan-Meier plot of overall survival comparisons between PD-1 inhibitors in patients with PD-L1 TPS≥1%.

1. Camrelizumab vs. Toripalimab; (B) Camrelizumab vs. Nivolumab; (C) Toripalimab vs. Nivolumab.

| (A)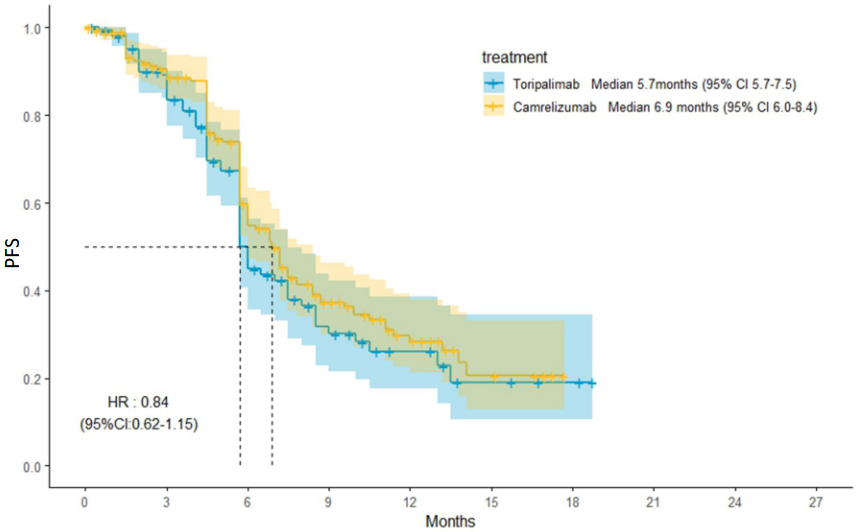 |
| --- |
| (B)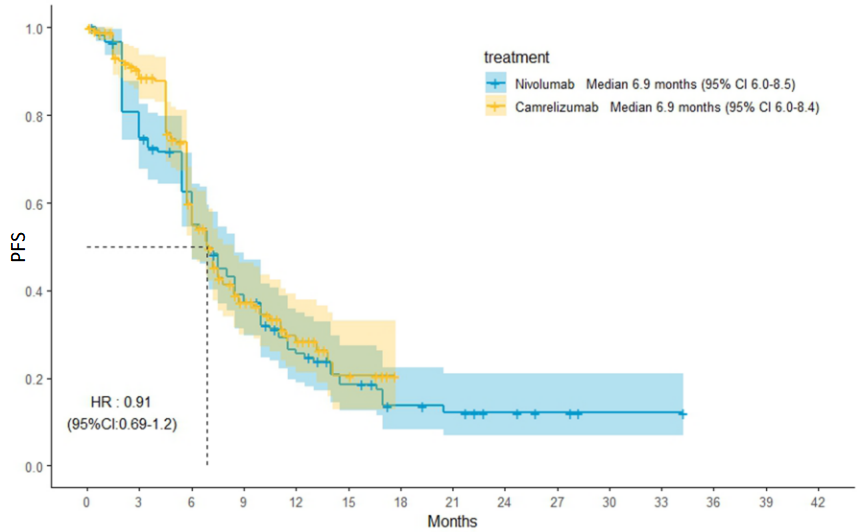 |
| (C)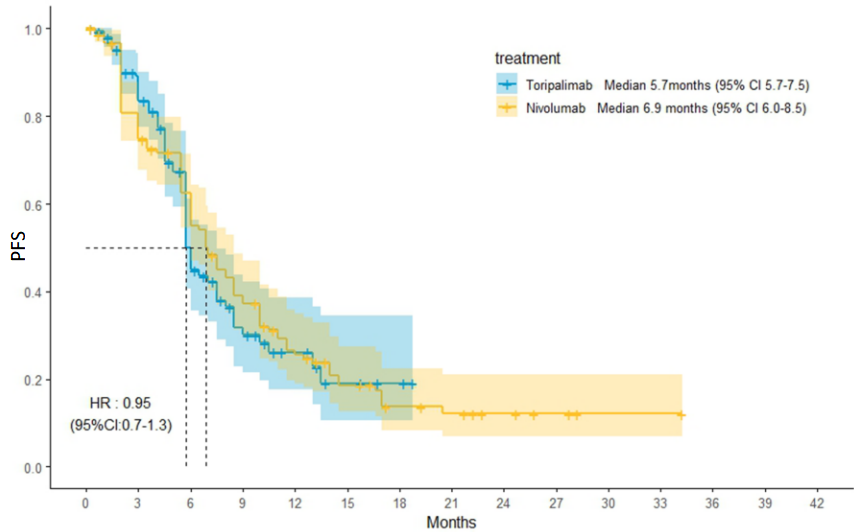 |

**Supplementary Figure S5.** Reconstructed Kaplan-Meier plot of progression-free survival comparisons between PD-1 inhibitors in patients with PD-L1 TPS≥1%.

1. Camrelizumab vs. Toripalimab; (B) Camrelizumab vs. Nivolumab; (C) Toripalimab vs. Nivolumab.

| (A)  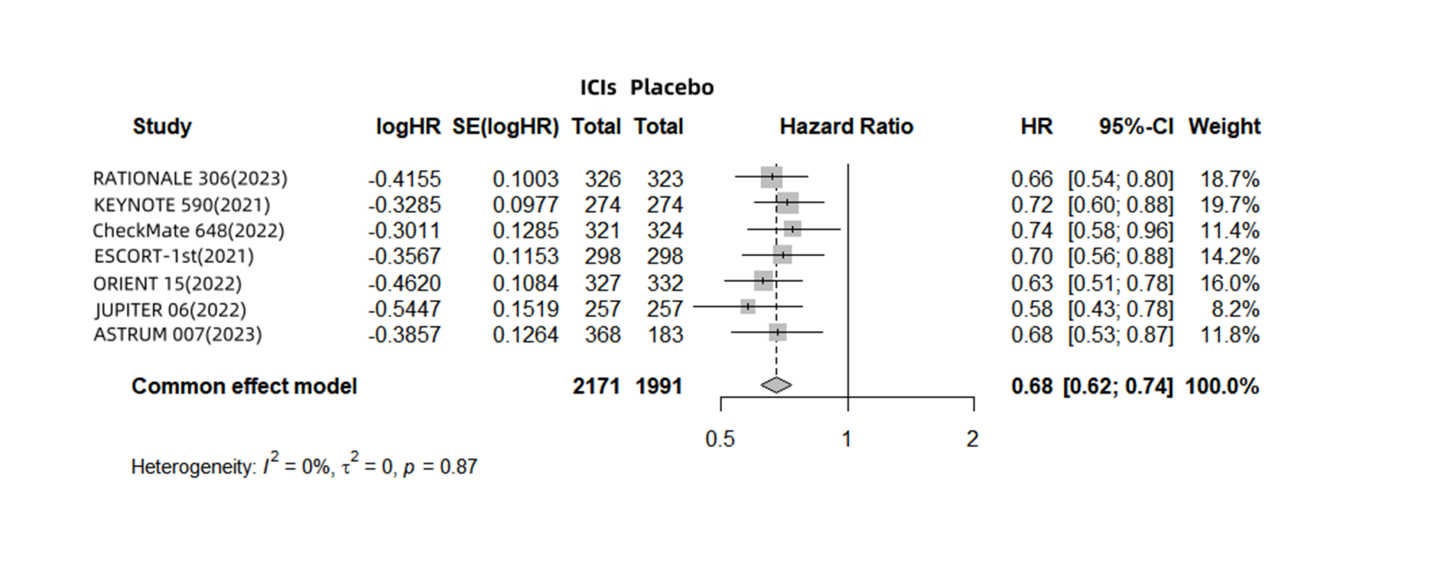 |
| --- |
| (B)  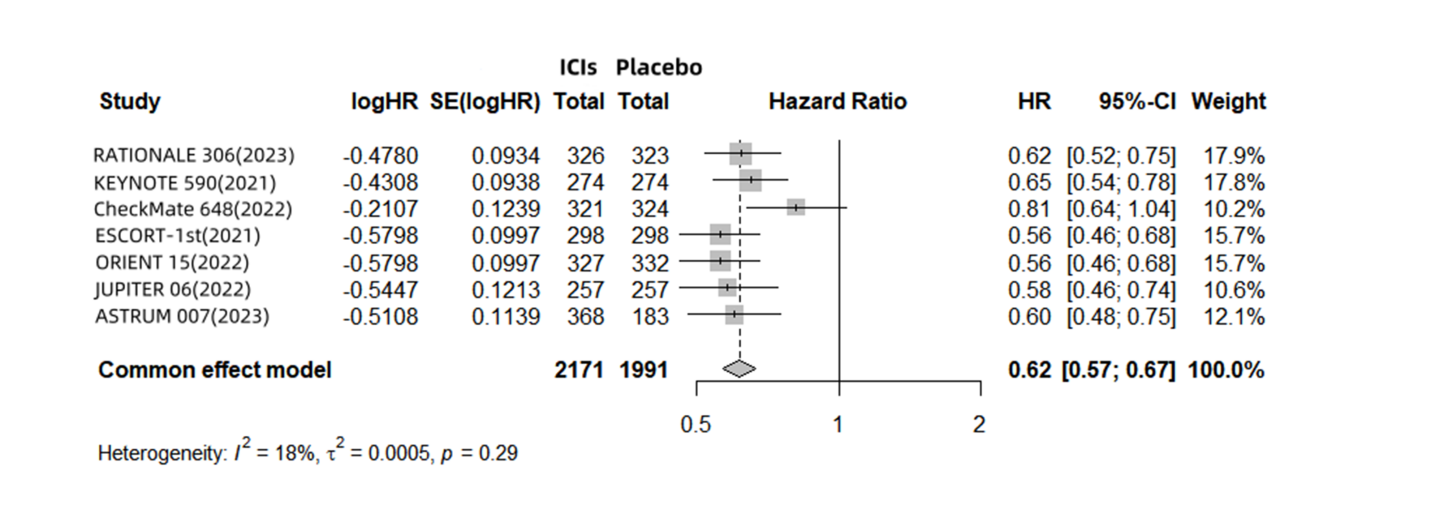 |

**Supplementary Figure S6.** Forest plot for (A) overall survival and (B) progression-free survival that compared PD-1 inhibitor plus chemotherapy with chemotherapy alone in esophageal cancer patients. ICIs, immune checkpoint inhibitors; HR, hazard ratio.

| (A)  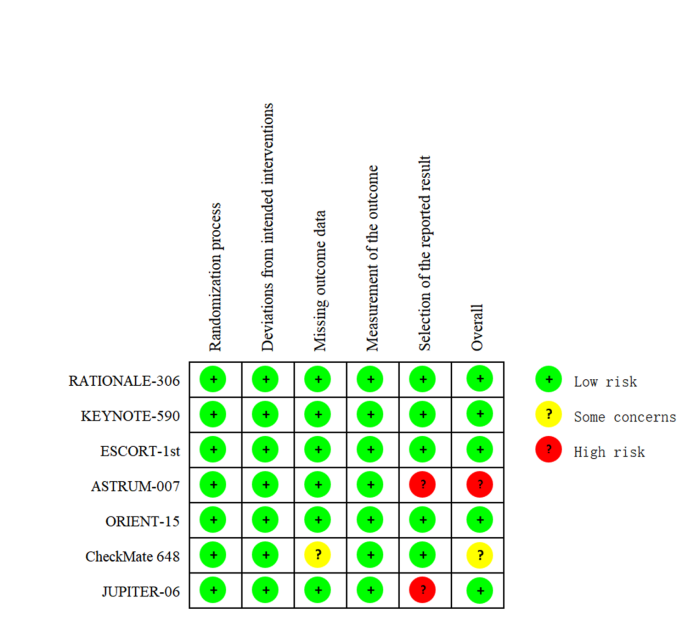 | (B)  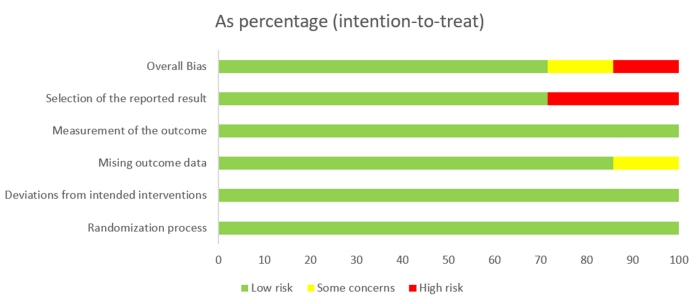 |
| --- | --- |

**Supplementary Figure S7.** Details of the bias risk assessment for each study.

| (A)  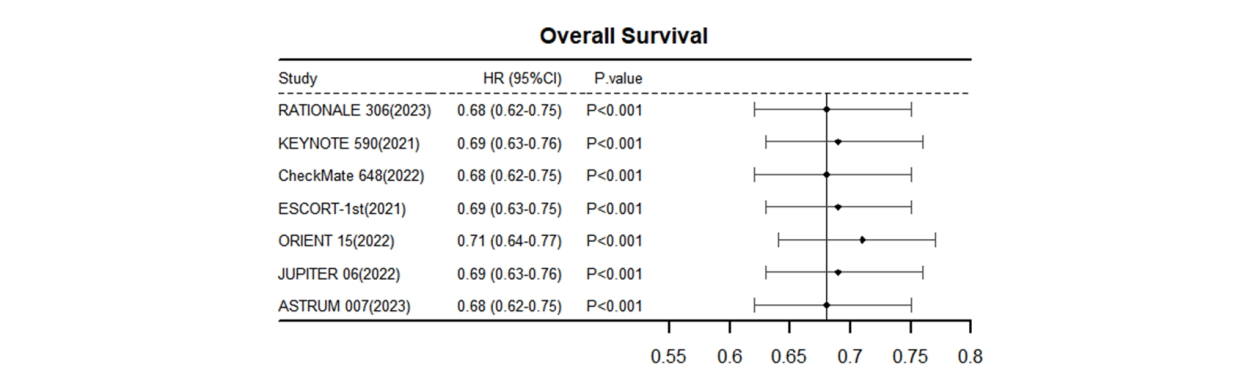 |
| --- |
| (B)  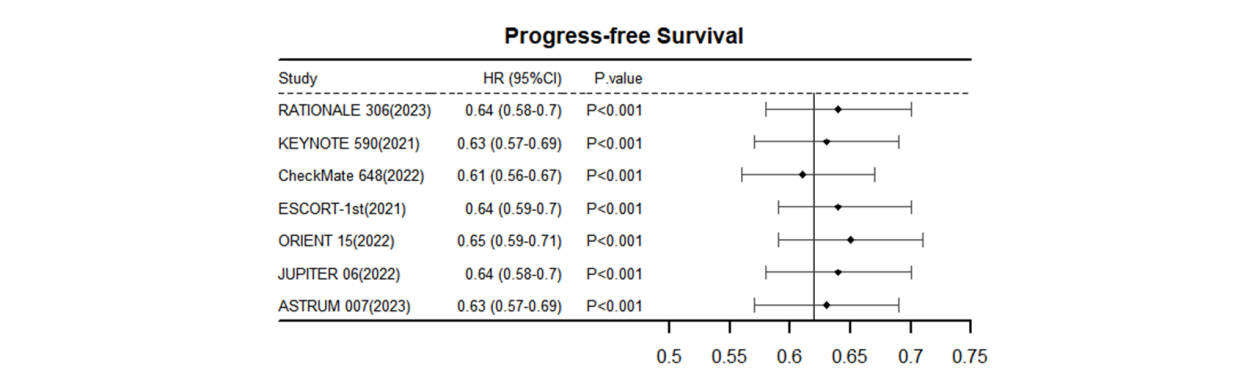 |

**Supplementary Figure S8.** Results of sensitivity analyses by excluding of the listed trials.

HR, hazard ratio.

**Supplementary Table S1.** Heterogeneity analysis in the meta-analysis of survival after synthesis.

|  | Q | H^2^ | I^2^(%) | higher limit of H value | lower limit of H value |
| --- | --- | --- | --- | --- | --- |
| Pooled overall survival for ICIs | 144.80 | 0.70 | 0 | 0.86 | 0.82 |
| Pooled overall survival for placebo | 138.47 | 0.71 | 0 | 0.86 | 0.83 |
| Pooled progression-free survival for ICIs | 211.51 | 1.09 | 8.75 | 1.05 | 1.04 |
| Pooled progression-free survival for placebo | 164.71 | 0.97 | 0 | 0.99 | 0.98 |

ICIs, immune checkpoint inhibitors.

**Supplementary Table S2.** Subgroup analysis of overall survival and progression-free survival.

| **Study subgroup** | **Overall survival** | | | **Progression-free survival** | | |
| --- | --- | --- | --- | --- | --- | --- |
|  | **No. of studies** | **Pooled hazard ratio (95% CI)** | **Meta-pregression, P-value** | **No. of studies** | **Pooled hazard ratio (95% CI)** | **Meta-pregression, P-value** |
| **Total** | 7 | 0.68 [0.62-0.74] |  | 7 | 0.62 [0.57-0.67] |  |
| **Sex** |  |  | 0.44 |  |  | 0.37 |
| Male | 6 | 0.66 [0.59-0.72] |  | 4 | 0.56 [0.50-0.63] |  |
| Female | 6 | 0.73 [0.56-0.95] |  | 4 | 0.66 [0.48-0.90] |  |
| **Age, years** |  |  | 0.14 | 4 |  | 0.67 |
| ≤ 65 | 6 | 0.70 [0.63-0.79] |  | 4 | 0.60 [0.52-0.68] |  |
| > 65 | 6 | 0.61 [0.54-0.70] |  | 4 | 0.57 [0.47-0.68] |  |
| **Race** |  |  | 0.69 |  |  |  |
| Asian | 6 | 0.67 [0.61-0.73] |  | 4 | 0.57 [0.52-0.64] | 0.96 |
| Non-Asian | 2 | 0.71 [0.55-0.90] |  | 0 | NA |  |
| **ECOG PS** |  |  | 0.34 |  |  | 0.48 |
| 0 | 6 | 0.62 [0.53-0.74] |  | 4 | 0.53 [0.42-0.66] |  |
| 1 | 6 | 0.69 [0.62-0.76] |  | 4 | 0.58 [0.52-0.66] |  |
| **Disease status at trial entry** |  |  | 0.76 |  |  | 0.76 |
| Metastatic | 6 | 0.64 [0.58 0.71] |  | 4 | 0.57 [0.51-0.64] |  |
| Locally advanced | 5 | 0.61 [0.46-0.81] |  | 4 | 0.60[0.45-0.80] |  |
| **Chemotherapy** |  |  | 0.68 |  |  | 0.63 |
| paclitaxel plus cisplatin | 4 | 0.66 [0.59-0.75] |  | 3 | 0.56 [0.0-0.63] |  |
| fluorouracil plus cisplatin | 4 | 0.69 [0.59-0.80] |  | 2 | 0.60 [0.48-0.74] |  |

ECOG PS, Eastern Cooperative Oncology Group performance score.
